# Convergent morphology and divergent phenology promote the coexistence of *Morpho* butterfly species

**DOI:** 10.1038/s41467-021-27549-1

**Published:** 2021-12-13

**Authors:** Camille Le Roy, Camille Roux, Elisabeth Authier, Hugues Parrinello, Héloïse Bastide, Vincent Debat, Violaine Llaurens

**Affiliations:** 1 Institut de Systématique, Evolution, Biodiversité (ISYEB), Muséum National d’Histoire Naturelle, CNRS, Sorbonne Université, EPHE, Université des Antilles, CP50, 75005 Paris, France; 2 Sorbonne Paris Cité, Université Paris Descartes, 12 rue de l’École de Médecine, 75006 Paris, France; 3 Department of Experimental Zoology, Wageningen University, 6709 PG Wageningen, The Netherlands; 4 CNRS, UMR 8198 - Evo-Eco-Paleo, Univ. Lille, F-59000 Lille, France; 5 MGX-Montpellier GenomiX, Univ. Montpellier, CNRS, INSERM, F-34094 Montpellier, France; 6 CNRS, IRD, UMR Évolution, Génomes, Comportement et Écologie, Université Paris-Saclay, 91198 Gif-sur-Yvette, France

**Keywords:** Behavioural ecology, Speciation, Evolutionary ecology

## Abstract

The coexistence of closely-related species in sympatry is puzzling because ecological niche proximity imposes strong competition and reproductive interference. A striking example is the widespread wing pattern convergence of several blue-banded *Morpho* butterfly species with overlapping ranges of distribution. Here we perform a series of field experiments using flying *Morpho* dummies placed in a natural habitat. We show that similarity in wing colour pattern indeed leads to interspecific territoriality and courtship among sympatric species. In spite of such behavioural interference, demographic inference from genomic data shows that sympatric closely-related *Morpho* species are genetically isolated. Mark-recapture experiments in the two most closely-related species unravel a strong temporal segregation in patrolling activity of males. Such divergence in phenology reduces the costs of reproductive interference while simultaneously preserving the benefits of convergence in non-reproductive traits in response to common ecological pressures. Henceforth, the evolution of multiple traits may favour species diversification in sympatry by partitioning niche in different dimensions.

## Abstract (web summary)

‘Here, the authors use dummies of different *Morpho* butterfly species and sexes to investigate behaviour in patrolling butterflies, finding strong reproductive interference between species despite limited gene flow. They finally show that interference is mitigated by temporal partitioning, hence promoting the coexistence of sympatric *Morpho* species.’

## Introduction

Natural communities are composed of multiple species involved in diverse ecological interactions either facilitating or impairing their coexistence. These interactions strongly depend on the level of phylogenetic divergence between the involved species^1,2^. In particular, the coexistence of closely-related species is often impaired by their ecological and morphological similarities^3–5^, generating either elevated interspecific competition and/or reproductive interference^6–8^. Close relatedness and shared phenotypes are indeed identified as important factors enhancing behavioural interference in sympatric animal species^9^. Nevertheless, inherited traits shared by closely-related species may also provide individual fitness benefits, for instance when facing common predators either by sharing a common warning signal^10,11^, or by responding to alarm cues emitted by heterospecifics^12,13^. Such interspecific positive density-dependence is predicted to favour species coexistence^14^. Here, we investigate how shared and divergent traits can facilitate the coexistence of closely-related species.

Mimetic butterflies are a striking case of sympatric species with phenotypic convergences strongly promoted by natural selection. The convergence observed for warning wing patterns in defended species is frequently followed by convergence in other traits like flight height^15,16^ or host-plant^17^ since sharing a microhabitat increases the similarity of encountered predatory communities, thereby enhancing protection^18^. Nevertheless, the benefits conferred by overlapping visual signals and ecological niches in mimetic species may, in turn, incur fitness costs through increased heterospecific rivalry, heterospecific female harassment and the expression of Dobzhansky-Müller incompatibilities in hybrids^19–21^. These costs might ultimately either limit the coexistence of closely-related mimetic species^22^ or promote the evolution of alternative cues involved in species recognition^23^. The conflicting ecological interactions between mimetic species, therefore, question their persistence in sympatry when they are closely related and point to the evolution of alternative divergent traits.

Here we investigate how trait evolution might favour the coexistence of closely-related species by focusing on three species of the butterfly genus *Morpho* that are sympatric over most of the Amazonia^24,25^ and occupy the same understory niche in the tropical forest. Multiple local convergences in wing colour patterns are observed among these species, throughout their geographical range^25^. Although not chemically defended, these butterflies are very difficult to capture because of their fast, erratic flight. The contrast between their dorsal bright iridescent blue and ventral cryptic brownish wing surfaces induces a flash pattern during flapping flight, that was suggested to confuse predators, further increasing their difficulty of capture^26^. Predators may then learn to avoid such elusive prey harbouring the conspicuous blue patterns. The local convergence in the three closely-related *Morpho* species probably stems from frequency-dependent selection generated by predator behaviour, in a similar way as in Müllerian mimics^25^. The iridescent blue colouration of *Morpho* butterflies shared by sympatric species may thus reduce individual predation by advertising escape ability (the ‘escape mimicry hypothesis’, see ref. ^27^). Males and females typically display the same colour pattern shared between sympatric species and may thus benefit from increased protection against the same predator community. While females are rarely observed in the field, males from these different closely-related species typically patrol within the same habitats. Male–male interactions are then frequently observed in these butterflies, one of the males eventually being chased away. Male territoriality may therefore occur both within and among species, potentially leading to important reproductive interferences^28^. The local convergence in colour pattern might further enhance heterospecific rivalry and impair mate recognition in these sympatric species. The persistence of closely-related species sharing similar colouration would thus be enabled if intraspecific benefits of maintaining a convergent colour pattern outweigh the costs associated with reproductive interference.

To test whether convergent wing patterns generate reproductive interference between species, we investigated the behaviour of *Morpho* towards flying dummy butterflies harbouring various wing patterns, in a series of experiments performed in the wild. To infer whether these heterospecific reproductive behaviours underpin genetic exchange, we then estimated the gene flow among them, their level of genomic divergence and investigated their demographic history. To explore whether interference could be mitigated by temporal partitioning, we finally monitored male flight activity using capture-mark-recapture experiments.

Our study reveals strong genetic isolation between species despite reproductive interference, probably facilitated by a marked difference in flying hours between *Morpho* species. This work highlights how joint convergent and divergent evolution of different traits may promote species diversification in sympatry.

## Results and discussion

We focused on a single locality from Amazonian Peru where three *Morpho* species with strikingly similar colour patterns (*Morpho achilles*, *Morpho helenor* and *Morpho deidamia*), live in sympatry. In this field site, males from these three closely-related species display a typical patrolling behaviour along the river bed, allowing us to investigate species interactions *in natura*.

### Heterospecific interactions lead to reproductive interference among sympatric species

**Fig. 1:**
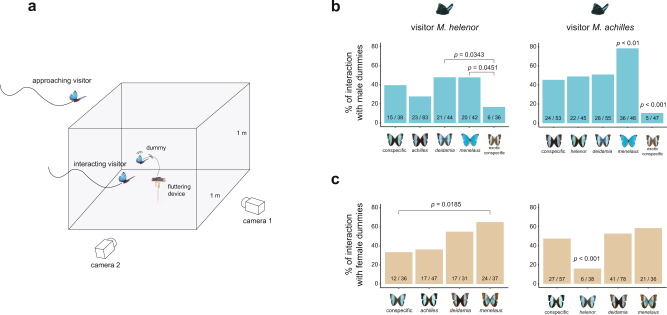
Behaviour of *Morpho* towards flying dummy butterflies of conspecifics and congeners. **a** Experimental set up used to study flight interaction. The dummy butterfly was placed at the centre of a cubic area materialised by four 1 m^3^—sticks, and fixed to a solar-powered fluttering device reproducing butterfly flying behaviour. Interaction between the visitor and dummy butterfly (defined as a visitor entering in the cubic area) were recorded using a stereoscopic high-speed videography system, allowing to quantify flight trajectory during the interaction (Fig. 2). **b** Frequency of interaction with conspecific and congener male dummies (blue colour) in the two mimetic sister species *Morpho helenor* (left column) and *M. achilles* (right column). **c** Frequency of interaction with the female dummies (yellow colour). Raw data << nb of interactions/nb of approaches >> are indicated on each bar. Proportions were compared using Fisher Exact probability tests. Only significant differences are shown (*P* < 0.05). For proportions differing from all the others, the highest *P*-value is reported. Source data are provided as a Source Data file.

To test whether the strong convergence in colour pattern among these sympatric *Morpho* species leads to heterospecific rivalry and courtship, we investigated the response of patrolling males to butterfly dummies placed in the field. We built realistic moving butterfly dummies using the actual wings of captured *Morpho*, set up on a solar-powered fluttering device (Fig. 1a and Supplementary Video 1; see also Supplementary Figs. 1, 2). To ensure that only visual cues were triggering the interactions, the wings were washed in hexane prior mounting, ruling out any effect of pheromones^29^. We built ten different dummies with the wings of specimens from different species and sexes. Eight dummies were built from butterflies caught in the same Peruvian site, using both sexes of four sympatric species: the three mimetic species *M. helenor*, its sister species, *M. achilles*, and the more distantly-related *M. deidamia*, all exhibiting an iridescent blue band bordered by proximal and distal black areas, and the phenotypically distinct *Morpho menelaus*, exhibiting fully blue iridescent wings (referred to as local dummies: *n* = 8) (Fig. 1b, c and Supplementary Fig. 1). The last two dummies were built from *M. helenor* and *M. achilles* males captured in French Guiana (referred to as exotic dummies: *n* = 2), exhibiting a narrower blue band relative to local—Peruvian—individuals (Fig. 1b and Supplementary Fig. 1). We tested all dummies (*n* = 10) following a randomised design: a different dummy was placed each morning at the same site and left fluttering on the river bank for 5 h. This was replicated four times per dummy. The dummy was continuously filmed using a camera (Gopro Hero5 Black set at 120 images per second) and monitored by a human observer who recorded the timing of responses displayed by wild butterflies.

Over a 2-months period, we recorded the patrolling behaviours of all males passing through the river on sunny mornings, resulting in 2700 responses to dummies. We specifically focused on the behaviour of butterflies from the two mimetic sister species *M. helenor* and *M. achilles*, which represented the large majority of the passing males (35% and 47% respectively, Supplementary Fig. 3). Visiting species were identified by sight, except for *M. helenor* and *M. achilles* that cannot be reliably discriminated in flight. We used an indirect method to distinguish these two strikingly similar species (see ‘Methods’ section and Supplementary Fig. 13). During these sessions, we defined two behaviours: (1) approaches, when a marked change in the trajectory of the passing butterfly towards the dummy was observed and (2) interactions, when the butterfly entered a 1 m^3^ zone around the dummy (Fig. 1a). We controlled for the effect of cloud cover on these behaviours (see ‘Methods’ section and Supplementary Fig. 4). This procedure allowed us to test whether patrolling *Morpho* males (1) are more strongly attracted by the colour pattern of their conspecifics as compared to that of other species, (2) discriminate between sexes and (3) are more attracted by local colour patterns than by exotic ones, thereby assessing the strength of behavioural interference between sympatric species.

About half of the patrolling individuals deviated from their flight path to *approach* the setup (see Supplementary Figs. 5, 6 and Supplementary Tables 2–4 for more details). Wing area and proportion of iridescent blue on the dummy wings had limited effect on the percentage of *approach* (Supplementary Fig. 7 and Supplementary Table 7): the long-range blue signal emitted by the different wing patterns displayed in the different species thus appeared similarly attractive to patrolling *M. helenor* and *M. achilles* males, suggesting they may approach anything roughly recognised as a potential mate or rival.

In both *M. helenor* and *M. achilles*, about 40% of *approaches* resulted in interactions, the visitor typically flying in circles around the dummy (Supplementary Video S1). Surprisingly, interactions with conspecific dummies were not significantly higher than with sympatric heterospecifics. Overall, no strong discrimination between sympatric conspecifics and sympatric heterospecifics, nor between female and male dummies was found, suggesting that interspecific interactions are frequent among wild *Morphos* in sympatry (Fig. 1b, c). Some differences were nevertheless detected. Dummies with a larger wing area and a greater proportion of iridescent blue colouration were more likely to trigger interactions (Supplementary Fig. 7), suggesting that these characteristics are used by patrolling males to discriminate encountered individuals. *M. helenor* males were found to approach dummies (all sexes and species pooled) significantly more than *M. achilles* males (mean % of approach in *M. helenor* = 48.3 ± 15.7; *M. achilles* = 39.7 ± 14.8; *P* = 0.01). They interacted as frequently with *M. achilles* females as with their conspecific females (Fig. 1b). In contrast, interactions occurred in markedly lower proportions with the exotic dummies, that were largely ignored (Fig. 1b). *Morpho* males, therefore, do distinguish different colour patterns (i.e. large blue band in local individuals, vs. narrower blue band in exotic ones), but yet they largely engage in *interactions* with congeners bearing locally known signals, including signals sharply dissimilar from their own displayed by more distantly-related species (e.g. fully blue wings in *M. menelaus*). For males, interspecific interactions with both males and females could be promoted because mating opportunities are limited by strong male–male competition. Defending territory against any males harassing a potential mate, or courting any females would then be a favoured behaviour. The costs of missed mating opportunities are probably elevated for males when species recognition is impaired by the phenotypic resemblance between species^10^.

Overall, the strong visual attraction and the low species discrimination suggest that both heterospecific interactions—contests among males and mating or mating attempts with females—can frequently happen. This indicates that reproductive interferences occur among these sympatric *Morpho* species.

Patrolling *M. achilles* males had limited interactions with the co-mimetic *M. helenor* female, but interacted indiscriminately with the dummies of the other sympatric species (i.e. *M. deidamia* and *M. menelaus*) (Fig. 1c). Such a specific, acute visual discrimination towards its —albeit phenotypically closest—sister species possibly evolved in *M. achilles* as a result of reinforcement selection against genetic incompatibilities expressed in hybrids^30^. Whether reinforcement selection does occur in those closely-related species would however require further study. Reinforcement process may also occur through divergence in olfactory cues enabling discrimination among species^20,31^, although this remains to be investigated in *Morpho*.

**Fig. 2:**
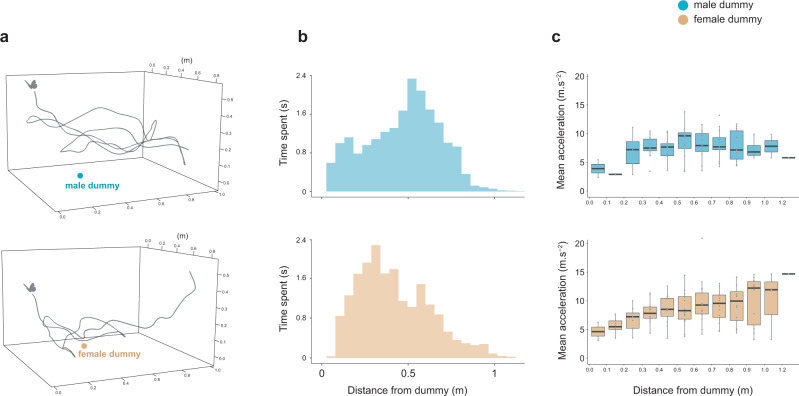
Three-dimensional kinematic of contest and courtship flights. **a** Example of a flight path when circling around the dummy conspecific male (top) and female (bottom). **b** Variation in time spent as a function of distance from the dummy. **c** Variation in flight acceleration as a function of distance from the dummy. Boxplots show the median and inter-quartile range (IQR), while whiskers depict the data range (75th and 25th ± 1.5*IQR, respectively). *N* = 14 flight interactions analysed with the dummy male and 14 with the dummy female. Source data are provided as a Source Data file.

This specific effect detected in female interactions suggests that patrolling males can discriminate sexes based on short-distance visual cues. To test whether the behaviour of *M. achilles* males indeed differs when interacting with conspecific male and female dummies, we equipped our set up with a stereoscopic high-speed videography system, enabling us to quantify the three-dimensional flight kinematics of visiting butterflies in natural conditions on a sub-set of sessions (Figs. 1a and 2a). Behavioural differences were detected between the interactions toward males and females (*n* = 14 flights analysed for each sex): wild males circled closer to the female compared to the male dummy (significant interaction between distance from dummy and the sex of the dummy on the proportion of time spent within distance intervals: *F*_11,312_ = 1.9; *P* = 0.03, Fig. 2b, see also Supplementary Fig. 8), ensuring a steady speed when close to the female, whereas they displayed more erratic accelerations around the male dummy (significant interaction between the distance from dummy and the sex of the dummy on acceleration within distance intervals: *F*_1,177_ = 1.9; *P* < 0.001, Fig. 2c). These aerial interactions suggest that *M. achilles* males adjust their flight behaviour according to the sex of their conspecific, showing that, at least in *M. achilles*, *Morpho* males can identify females using visual cues alone.

The limited attraction of *M. achilles* towards the females of its sister species *M. helenor* and the contrasted attraction to local and exotic colour patterns suggest that sympatric interactions might have promoted discrimination capacities in the closest related species, thereby reinforcing pre-zygotic barriers. Overall, the behavioural interferences among these sympatric *Morpho* species as well as the putative reinforcement effect detected in *M. achilles* question their stable coexistence and reproductive isolation.

### Limited genetic exchanges between species despite close relatedness and reproductive interference

**Fig. 3:**
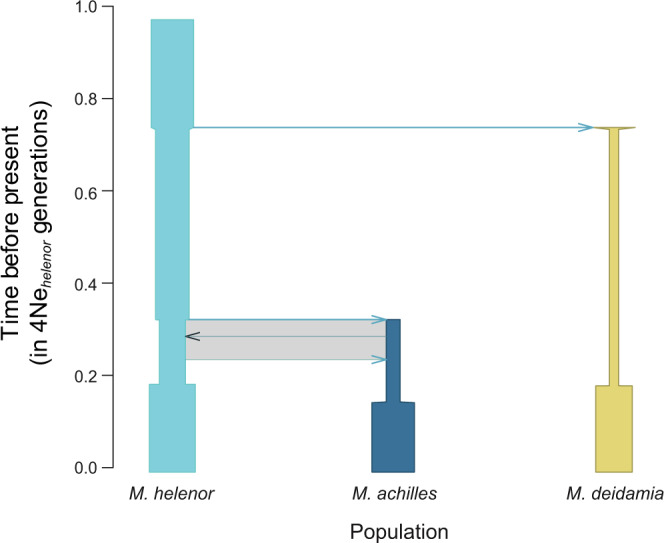
Best inferred demographic scenario from RAD-sequencing data. Eight categories of scenarios were compared according to different temporal patterns of gene flow: between *M. helenor* and *M. achilles* (ancestral migration or secondary contact); with *M. deidamia* (strict isolation, migration only with *M. helenor*, migration only with *M. achilles*, migration between the three species). The parameter values of the best demographic model are expressed relative to the current population size of *M. helenor* set to Ne = 1. The grey rectangle indicates the period when *M. helenor* and *M. achilles* were, according to the demographic model fitting the best the molecular dataset, genetically connected through migration event.

To test the level of reproductive isolation despite such a strong behavioural interference, we then used genomic data obtained for the three mimetic species *M. achilles*, *M. helenor* and *M. deidamia*. Patterns of genetic polymorphism (Supplementary Figs. 9 and 10) and divergence (Supplementary Fig. 11) throughout the genome were investigated using RAD-sequencing. Eight categories of contrasted scenarios of speciation and models of linked selection (Supplementary Fig. 12) were then proposed to be statistically evaluated using a modified version of DILS 1.0.0 adapted to three gene pools^32^. Each category was simulated using four sub-models depending on whether the effective population size is assumed to be homogeneous or heterogeneous along the genome, as well as for introgression rates. Consistent with the phylogeny^33^, demographic inferences performed on our Peruvian populations revealed a more recent time of the split between the sister species *M. achilles* and *M. helenor* than between these two species and *M. deidamia*. Our hierarchical approach of eight categories of models according to temporal patterns of migration between all three species provided strong statistical support for current genetic isolation between *M. deidamia* and both *M. helenor*/*M. achilles* (*F*_st_ ± std *M. helenor* – *M. achilles* = 0.30 ± 0.226; *M. helenor* – *M. deidamia* = 0.82 ± 0.11; *M. achilles* – *M. deidamia* = 0.85 ± 0.11) (Fig. 3; posterior probability = 0.94). Current isolation was also strongly supported between *M. helenor* and *M. achilles*, with gene flow restricted to the early times of speciation (posterior probability = 0.80). Genomic or behavioural barriers are thus likely to strongly limit gene flow between these closely-related sympatric species (Fig. 3 and Supplementary Tables 8–10). Note that if our demographic inferences show that models with ancestral migration reproduce the molecular data better than models with ongoing introgression, we cannot rule out the possibility that more complex, untested models might provide a better fit to the data (see ‘Methods’ section). This inferred strict genetic isolation despite close relatedness and reproductive interference between *M. achilles* and *M. helenor* nevertheless suggests that ecological factors might prevent gene flow in sympatry.

### Temporal segregation between sympatric sister species

**Fig. 4:**
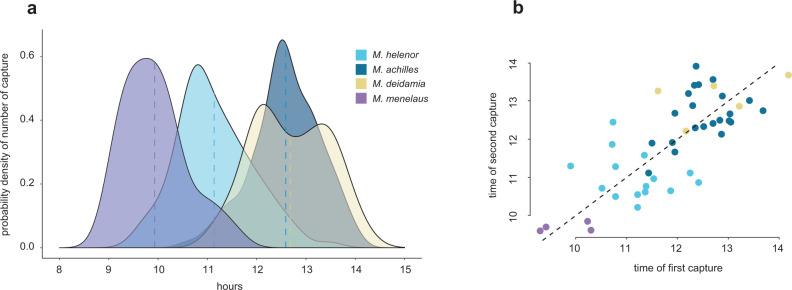
Patrolling time among sympatric species and individuals. **a** Segregation of patrolling time throughout the day among the different blue *Morpho* species was observed in sympatry. Dashed vertical lines indicate the mean flight time. **b** Consistency of patrolling hours within individuals estimated by the correlation between the time of first and second capture within the sub-sample of recaptured individuals. The dashed line represents exact same time between the first and second capture. Source data are provided as a Source Data file.

The analysis of the temporal variations of flight activity in a mark-recapture experiment revealed a striking difference in patrolling time among species (Kruskal–Wallis test: Chi-square = 179.7, *P* < 0.001, df = 3), with little overlap between the sister species *M. achilles* and *M. helenor* (Fig. 4). Males *M. helenor* patrolled earlier than *M. achilles* (mean patrolling time ± s.d. = 11:14 ± 00:45 vs. 12:35 ± 00:40, respectively). Patrolling time in *M. deidamia* (12:40 ± 00:46) however overlapped with *M. achilles*. Time of (re)capture of the same individual in *M. achilles* was notably stable (correlation between time of first *vs*. second capture: *r* = 0.40; *P* = 0.05), suggesting a regularity in patrolling time at the individual level in this species (Fig. 4). Whether such individual temporal regularity is genetically determined or reflects a plastic behaviour^34,35^ remains to be investigated.

Reproductive and/or aggressive interference are generally expected to promote spatial or temporal habitat segregation between species because it reduces the cost of negative interspecific interactions^28,36^. The observed shift in flight activity peaks suggests that reproductive interferences among the morphologically convergent *Morpho* species have favoured the evolution of divergent temporal niches in *M. achilles* and *M. helenor*. Remarkably, this temporal segregation extended to the more distantly-related *Morpho menelaus*, that flies earlier in the morning (Fig. 4). In particular, no overlap between its activity range and that of *M. achilles* was detected, mitigating the potentially strong interference between these species suggested by the attraction of patrolling *M. achilles* towards *M. menelaus* dummy (Fig. 1b). Whether the temporal segregation observed between these sympatric *Morpho* species was involved in a sympatric speciation process or stem from reinforcement after secondary contact between these closely-related species remains to be elucidated, as well as the genetic vs. plastic origin of this behavioural trait.

Temporal segregation in response to resource competition or reproductive interference has been reported in other taxa^37–39^, but mostly at a larger temporal scale (e.g. seasonal segregation). Peaks of patrolling activity between sister *Morpho* species are shifted by less than 2 h, representing a remarkably fine temporal scale for a shift in reproductive behaviour^39^. Nonetheless, comparable fine-scale temporal partitioning of sexual activity has been observed in other moth and butterfly species^35,40^, suggesting that it may be relatively common in Lepidoptera.

Altogether, our results suggest that there is a potential for strong interspecific competition among males from *Morpho* species in sympatry, but that this competition is mitigated at least in part by their temporal segregation. Heterospecific courtships may also be limited, if the temporal segregation observed in *Morpho* males is mirrored by a similar temporal partitioning of females’ activities. This could not be tested here as females were almost never seen flying along the riverbank: future studies specifically exploring females behaviour should thus be performed. Synchronisation of mating activity between sexes is however likely^41,42^, because any deviation of males relative to females activity would reduce the probability of intraspecific mating^34,35^. In contrast, a shift in mating time among species would act as a powerful isolation mechanism^39^, explaining how co-mimetic *Morpho* species can maintain a similar colour pattern in the same habitat while remaining sexually isolated.

Divergence in daily phenology in butterfly mating activities may be a widespread process enabling the persistence of diversity-rich assemblages, as suggested by several reports of temporally structured sexual activities in other butterflies^43–45^, including closely-related species^46^. Our study shows that the coexistence of closely-related species can generate complex ecological interactions, both mutualistic (mimicry) and antagonistic (reproductive interference), that could be mitigated by shifts in temporal niches. The evolution of multiple traits, morphological and behavioural, may thus favour species diversification in sympatry by partitioning niche along different ecological dimensions.

## Methods

### Study site and population

The study was conducted between July and October 2019 in the North of Peru. We focused on populations of coexisting *Morpho* species present in the regional park of the Cordillera Escalera (San Martin Department) near the city of Tarapoto. Both the capture-recapture and the dummy experiment were performed at the exact same location, on the bank of the Shilcayo river (06°27′14.364″S, 76°20′45.852″W).

### DNA extraction and RAD-Sequencing

Thirty-one wild males caught on the study site were sequenced to perform population genomic analyses (*M. achilles—n* = 13, *M. helenor—n* = 10 and *M. deidamia—n* = 8*)*. DNA was extracted from each sample from a slice of the thorax, using Qiagen kit DNeasy Blood & Tissue. DNA quantification (using the microfluorimetric method) and quality controls (using electrophoresis and spectrophotometric method) were performed prior to sequencing. RAD-library preparation and sequencing were performed at the MGX-Montpellier GenomiX platform (Montpellier, France). DNA was digested with the Pst1 enzyme and the library was prepared according to Baird and Etter’ protocol^47^ in a slightly modified version. Paired-end RAD-sequencing was performed on a 2 lanes flow cell of an Illumina HiSeq2500 in a rapid mode so that reads (125 bp) were expected to be of high quality with no missing base (N content). We obtained 299 million sequences, comprising R1 and R2 reads for each sequenced fragment. Adapters were removed from the reads.

### Read quality control, alignment and dataset generation

Read quality was assessed with FastQC v0.11.9 (http://www.bioinformatics.babraham.ac.uk/projects/fastqc/). The per base sequence quality was high across all reads (no lower than 36 for R1 and 32 for R2) with an average quality score of 39 (40 being the maximum). Overall, FastQC highlighted the high quality of the sequencing data, allowing us to skip the step of read trimming.

The data were demultiplexed, assigning each sequence to its sample ID and the reads were aligned using Stacks V2.5 (http://catchenlab.life.illinois.edu/stacks/)^48,49^. Parameters were set following the 80% polymorphic (*r80*) loci rule, which only considers loci shared by at least 80% of the samples^50^. The optimised parameters are ‘max distance between stacks’ (inside each sample) and ‘number of mismatches between stacks’ (between samples). Every other parameter was kept to default values. After aligning all reads, we selected 2740 biallelic loci shared by all samples, including 88,889 SNPs in total. Each locus had a length of 463.12 bp on average (range [343; 908]). These loci are assumed to be evenly distributed throughout the genome but cover only a limited portion of the genome (around 0.5%). Datasets were stored in a VCF file (containing all the SNPs found in the alignment) and a fasta file (containing the two alleles found at every locus for each sample). To run DILS-ABC inferences, Stacks fasta files were converted to another fasta format compatible with DILS (https://github.com/CoBiG2/RAD_Tools).

### Demographic inferences

Eight categories of demographic models were compared, according to temporal patterns of introgression. This was done to answer two questions on gene flow in *Morpho*: (1) is there ongoing migration between *M. helenor* and *M. achilles*? (2) do *M. helenor* and/or *M. achilles* exchange alleles with *M. deidamia*? This was assessed by an ABC approach using a version of *DILS* adapted to samples of three populations/species^32^. Since Stacks does not report monomorphic RAD loci, the ABC analysis was conditioned in the same way, by excluding monomorphic loci from the simulations. Focusing on polymorphic loci may only limit our ability to estimate the absolute values of parameters (i.e. population sizes expressed in numbers of individuals, and ages of past events expressed in numbers of generations); nevertheless, this framework excluding monomorphic loci still allows reliable comparisons of models^51^ and estimations of relative parameter values, as performed to investigate the human history^51^.

A generalist model was studied (Supplementary Fig. 12). This model describes an ancestral population subdivided in two populations: the ancestor of *M. deidamia* and the common ancestor of *M. helenor/M. achilles*. The latter population was further subdivided into the three species/populations currently sampled. Each split event is accompanied by a change in demographic size, the value of which is independent of the ancestral size. In addition, given clear genomic signatures for recent demographic changes with largely negative Tajima’s *D*, we implemented variations for the effective sizes of the three modern lineages at independent times. Finally, migration can occur between each pair of species/populations. Migration affecting the *M. helenor/M. achilles* pair can either be the result of secondary contact after a period of isolation (ongoing migration), or of ancestral migration (current isolation) as in^50,52^.

As this model is over-parameterised, our general strategy is to investigate the above two questions by comparing variations of this generalist model. Thus, to test the gene flow between *M. helenor* and *M. achilles*, we compared two categories of models. (1) With random parameter values for all model parameters including the ongoing migration between *M. helenor* and *M. achilles* (gene flow resulting from a secondary contact between them); (2) as above, but with the migration between *M. helenor* and *M. achilles* set to zero after a randomly drawn number of generations following their split. An overlap between ‘current isolation’ and ‘ongoing migration’ models can occur when the transition time (from ancestral migration to current isolation forward in time for a ‘current isolation’ model; or from ancestral isolation to ongoing migration forward in time for an ‘ongoing migration’ model) tends towards the extreme values 0 or *T*_*split*_ *hel-ach* (Supplementary Fig. 12). To reduce this effect, the transition times were drawn in a Beta distribution with parameters (*α* = 5, *β* = 1) when migration has to be restricted to a past period, and in a Beta distribution with parameters (*α* = 1, *β* = 5) when migration is assumed to occur after a recent secondary contact.

When two broad categories of models are statistically compared, each category is represented by simulations performed under the four sub-models allowing or not allowing genomic heterogeneities for effective sizes (Ne) and for migration rates (N.m). For instance, to test for gene flow between *M. helenor* and *M. achilles*, the model of ‘ongoing migration’ is actually represented by simulations with the four possible combinations of homogeneity/heterogeneity, all labelled as being ‘ongoing migration’.

As for any inferential analysis, it is important to recognise that the best-supported model is based on a classification of models within a studied set. Intermediate models, with more subtle cycles of genetic isolation and secondary contact could produce a better fit to the data, but it would be surprising to detect a strong support for the model assuming a lack of recent gene flow, if the most recent secondary contact of such cyclicity induced elevated gene flow.

For each model, 50,000 simulations using random combinations of parameters were performed. Parameters were drawn from uniform prior distributions. Population sizes were sampled from the uniform prior [0–1,000,000] (in diploid individuals); the older time of split was sampled from the uniform prior [0–8,000,000] (generations); ages of the subsequent demographic events were sampled in a uniform prior between 0 and the sampled time of split. Migration rates 4.N.m were sampled from the uniform prior [0–50]. Both migration rates and effective population sizes are allowed to vary throughout the genomes as a result of linked selection, following refs. ^53–55^.

On each simulated dataset, we calculated a vector of means and standard deviations for different summary statistics: intraspecific statistics (π for *M. helenor*, π for *M. achilles*, π for *M. deidamia*, θ_W_ for *M. helenor*, θ_W_ for *M. achilles*, θ_W_ for *M. deidamia*, Tajima’s *D* for M. helenor, Tajima’s *D* for *M. achilles*, Tajima’s *D* for *M. deidamia*) and interspecific statistics (gross divergence, net divergence and F_ST_ for all three possible pairs; ABBA-BABA *D*). Our version of DILS includes part of the DaDi^56^ and Moments^57^ strategy involving the identification of the best model proposed demographic model from the molecular patterns of polymorphism and divergence (proportion of shared polymorphisms, fixed differences between species, exclusive polymorphisms, etc.), excluding monomorphic loci. Thus, only loci containing at least one SNP in an alignment of the three species studied are considered, including singletons. Importantly, each locus carrying at least one SNP in a tri-specific alignment is associated with a mutation rate assumed to be 3 · 10^−9^ mutations per generation and per base pair to convert demographic parameters into demographic units from coalescence units.

We first conditioned the mutations occurring during coalescent simulations by using theta (=4 · *N* · *µ* · *L*_*i*_; where N is the effective population size, *µ* the mutation rate per nucleotide and per generation; *L*_*i*_ the length of locus *i*). The number of simulated segregating sites for a given locus strongly depends on the coalescent history (i.e the total length of the simulated coalescent tree), occasionally generating monomorphic loci. To confirm that the inferences are not impacted by differences in the number of monomorphic loci in the simulated datasets, we then used an alternative simulation approach, by randomly placing in simulated coalescent trees a fixed number of mutations corresponding to the observed number of SNPs for each locus. Thus, a randomly simulated dataset consists of 2740 loci whose lengths (ranging from 339 to 894 nucleotides) and number of SNPs (ranging from 1 to 91) individually match the properties of the observed loci in the actual dataset. Since the results drawn from both approaches were similar, we report only the estimations provided by the simulations based on the actual number of SNPs. Comparisons between the two approaches can be found in supplementary (Supplementary Tables 8, 9).

Statistical comparisons between simulated and observed statistics were performed using the R package abcrf version 1.8.1^58,59^.

### Mark-recapture experiment

To estimate the timing of patrolling activity among *Morpho* species, we performed capture-mark-recapture between 9 a.m. and 2 p.m. (flight activity in *Morpho* is drastically reduced in the afternoons at this site) during 17 sunny days. Although on a few days, capture was cancelled because of bad weather annihilating butterfly activity, the 17 capture sessions were mostly consecutives, as they were performed in a 22 days period (Supplementary Table 1 and Supplementary Fig. 15). All butterflies were captured with hand-nets, identified at the species level, and numbered on their dorsal wing surface using a black marker. The exact time of each capture was annotated. Butterflies captured while inactive, such as those laying on a branch or on the ground were excluded from the analysis to focus exclusively on actively patrolling individuals. We measured patrolling time for a total of 295 occasions, including 78 recaptures (i.e. 217 individuals were captured at least once). All captured individuals were males. Individuals *M. achilles* were the most frequently captured (*n* = 121), followed by *M. helenor* (*n* = 95). Individuals *M. deidamia* were about half less captured (*n* = 48), and individual *M. menelaus* were the least captured (*n* = 34). Because striking differences in patrolling time were observed among *Morpho* species, we used time of the day as a predictor of species identity in order to distinguish between *M. helenor* and *M. achilles* in the below-described experiment because butterflies from these two species are morphologically too similar to be identified while flying (Supplementary Fig. 13). After the 17 nearly-consecutive days of capture, one day of capture was repeated every 2 weeks during 2 months in parallel to the dummy experiment (described below), to verify that temporal activity was stable over time (Supplementary Fig. 13).

### Estimating population size from mark-recapture data

Based on capture-recapture histories, we estimated individual abundance for each species using a loglinear model implemented in the R package Rcapture version 1.4.3^60^ (Supplementary Fig. 15). Given the short duration the sampling period (22 days) relative to the longevity of adult *Morpho* butterflies (several months^61^), we used a closed-population model assuming no effect of births, deaths, immigration and emigration. Abundance was estimated in *Morpho helenor* and *M. achilles* only, as capture and recapture events were too few in the other species (*M. deidamia* and *M. menelaus*) to allow estimating population size (Supplementary Table 1).

### Experiment with dummy butterflies

We investigated the response of patrolling males to sympatric conspecifics, congeners and of exotic conspecifics, using dummies placed on their flight path. Dummies were built with real wings dissected and washed with hexane to remove volatile compounds and cuticular hydrocarbons, ensuring to test only the visual aspect of the dummies. We mounted the wings on a solar-powered fluttering device (Butterfly Solar Héliobil R029br) that mimics a flying butterfly, thereby increasing the attractiveness of the dummy. The fluttering dummy was positioned on the riverbank, and placed at the centre of a 1 m^3^ space delimitated with four vertical stacks (Fig. 1a). The set-up was continuously monitored by a human observer and filmed using a camera (Gopro Hero5 Black set at 120 images per second) mounted on a tripod. Patrolling *Morpho* butterflies that deviated from their flight path to approach the dummy but did not enter the cubic space were categorised as approaching. Any *Morpho* butterfly entering the cubic space was considered as interacting with the dummy. Those passing without showing interest to the setup were categorised as passing. The category of behaviour and the exact time of the butterfly responses were annotated on site by the human observer. Patrolling individuals were mainly identified at the species level by the observer on the site: *M. menelaus* can be easily distinguished from *M. deidamia*, and these two species are also quite different from *M. helenor* and *M. achilles*. However, the sister species *M. helenor* and *M. achilles* cannot be discriminated during flight, and we thus rely on an indirect method, based on flight hours, to infer the species identity of wild visitors looking as a *M. helenor*/*M. achilles* (Supplementary Fig. 13). Note that removing data with the highest levels of uncertainty in species identity (i.e. when discarding visits performed in the period where *M. helenor*/*M. achilles* temporally overlap) does not quantitively affect our results (Supplementary Fig. 14 and Supplementary Tables 5, 6). Using the recorded video, we also measured the duration of the interactions (i.e. the time spent in the cubic space) occurring between patrolling male and the dummy. The ten dummies were each tested during 4 sunny days from 9 a.m. to 2 p.m. (i.e. during 5 h). This resulted in 40 days of experiment over which each dummy was left fluttering on the river bank for a combined duration of 20 h. Dummies were randomly attributed to each day of the experiment. Mark-recapture data suggested a very low rate of individuals passing through the site several times per day (mean percentage of recapture within the same day = 0.95%), thus limiting potential pseudoreplication within each dummy replicate. We recently showed that intraspecific variation in wing colour pattern within the locality is very low in these species^25^. Using a single dummy per sex and species, as done here, should thus have little impact on the observed behaviours.

In order to control for variation in weather (affecting both the activity of patrolling butterflies and of the solar-powered device), we collected hourly data on the percentage of cloud cover for the period and location of our experiment (available at https://www.visualcrossing.com/). A percentage of cloud cover was then associated with all the behavioural observations, and used as a control variable in all statistical analyses.

### Three-dimensional kinematics of flight interaction with the dummies

To test whether *Morpho* males showed different flight behaviours when interacting with the male and female dummy, we filmed the flight interactions using two orthogonally positioned video cameras (Gopro Hero5 Black, recording at 120 images per second) around the dummy setup (Fig. 1a). Stereoscopic video sequences obtained from the two cameras were synchronised with respect to a reference frame (here using a clapperboard). Prior to each filming session, the camera system was calibrated with the direct linear transformation (DLT) technique^62^ by digitising the positions of a wand moved around the dummy. Wand tracking was done using DLTdv8^63^, and computation of the DLT coefficients was performed using easyWand^64^. After spatial and temporal calibration, we also used DLTdv8 to digitise the three-dimensional positions of both the visiting (real) butterfly and the dummy butterfly at each video frame by manually tracking the body centroid in each camera view. Butterfly positions throughout the flight trajectory were post-processed using a linear Kalman filter^65^, providing smoothed temporal dynamics of spatial position, velocity and acceleration of the body centroid. Based on these data, we investigated how spatial position, speed and acceleration of the visitor butterfly varied over the course of the interaction. We proceeded by dividing space into 10 cm spherical intervals around the dummy position ranging from 0 to 1.2 m distance (this step standardises interactions of different durations), and computed the proportion of time spent, the mean speed and acceleration of the interacting butterfly within each distance interval (Fig. 2). We analysed a total of 28 interactions performed by individual *Morpho achilles* male, including 14 with the dummy of its conspecific male and 14 with the dummy of its conspecific female. Analysed interactions lasted in average 1.44 ± 0.87 (mean ± sd) s.

### Statistical analysis of behavioural experiments

Differences in patrolling time were assessed by testing the effect of species on time of capture using Kruskal–Wallis test. To test the effect of visitor identity and dummy characteristics on the number of approaches and interactions, we performed logistic regressions. *Approach* was treated as a binary variable, where 0 meant ‘passing without approaching’ and 1 meant ‘approaching the dummy setup’. For the interactions, we only considered individuals approaching the setup, such as 0 meant ‘approaching without entering the cubic space’ and 1 meant ‘entering the cubic space’. This allowed getting rid of the uncertainties on whether passing individuals had actually seen the setup or not. We first tested the effect of visiting species on *approach* and *interaction* while controlling for dummy’s characteristics to test for intrinsic differences in territoriality (or ‘curiosity’) among species. We then tested the effect of the dummy sex and identity on *approach* and *interaction* separately in *Morpho helenor* and *M. achilles*. The percentage of cloud cover was also included in the models to control for variation in dummy movements (generated by the solar-powered device), potentially affecting the butterfly response (Supplementary Tables 3 and 4). We further tested if variation in wing area and proportion of iridescent blue among dummies affected the frequency of approach and interaction, again using logistic regression analyses (Supplementary Fig. 7). Statistical significance of each variables was assessed using likelihood ratio tests comparing logistic regression models^66^. Finally, we tested the effect of dummy sex and identity on the duration of interaction using Kruskal–Wallis tests.

Based on the flight kinematic data, we investigated whether flight behaviour during the interaction differed with male vs. female dummies. We ran a mixed-effects model testing the effect of (1) the sex of the dummy and of (2) the distance from dummy (fixed effects), on the proportion of time spent (fixed effects), using the flight ID as a random effect. The flight ID corresponds to the behaviour of a single wild males flying within the ‘interaction space’. Specifically, we tested for the statistical interaction between the sex of the dummy and distance from dummy on the proportion of time spent in the different distance intervals. We then similarly tested for difference in acceleration over the course of the flight interaction, by testing the effect of (1) the sex of the dummy and of (2) the distance from dummy (fixed effects), on the acceleration, with the flight ID as a random effect. We focused on the statistical interaction between the sex of the dummy and the distance from dummy on the mean acceleration in the different distance intervals.

### Reporting summary

Further information on research design is available in the Nature Research Reporting Summary linked to this article.

## Supplementary information

Supplementary Information

Description of Additional Supplementary Files

Supplementary Video 1

Reporting Summary

## Data Availability

All data supporting the findings of this study are provided as a Source Data file. The RNA-seq data generated in this study have been deposited to the National Center for Biotechnology Information under accession number PRJNA739839 (https://www.ncbi.nlm.nih.gov/). Source data are provided with this paper.

## Source data

Source Data

## Code availability

Genomic analyses were performed using the statistical analysis platform DILS (https://eep.univ-lille.fr/en/productions-2/dils-software/). A modified version of DILS adapted for studying divergence among three species/populations was used for this study. Source codes can be freely used from GitHub: https://github.com/Camille-Le-Roy/DILS_3pop_SNP [10.5281/zenodo.5707446].

## References

[1] Burns JH, Strauss SY (2011). More closely related species are more ecologically similar in an experimental test. Proc. Natl Acad. Sci. USA.

[2] Weber MG, Strauss SY (2016). Coexistence in close relatives: beyond competition and reproductive isolation in sister taxa. Annu. Rev. Ecol. Evol. Syst..

[3] Abrams P (1983). The theory of limiting similarity. Annu. Rev. Ecol. Evol. Syst..

[4] Graves GR, Gotelli NJ (1993). Assembly of avian mixed-species flocks in Amazonia. Proc. Natl Acad. Sci. USA.

[5] MacArthur R, Levins R (1967). The limiting similarity, convergence, and divergence of coexisting species. Am. Nat..

[6] Brown WL, Wilson EO (1956). Character displacement. Syst. Zool..

[7] Grant PR, Grant BR (2006). Evolution of character displacement in Darwin’s finches. Science.

[8] Gröning J, Hochkirch A (2008). Reproductive interference between animal species. Q. Rev. Biol..

[9] Losin N, Drury JP, Peiman KS, Storch C, Grether GF (2016). The ecological and evolutionary stability of interspecific territoriality. Ecol. Lett..

[10] Müller F (1879). Ituna and Thyridia: a remarkable case of mimicry in butterflies. Trans. Entomol. Soc. Lond..

[11] Sherratt TN, Beatty CD (2003). The evolution of warning signals as reliable indicators of prey defense. Am. Nat..

[12] Chivers D, Mirza R, Johnston J (2002). Learned recognition of heterospecific alarm cues enhances survival during encounters with predators. Behaviour.

[13] Dalesman S, Rundle SD (2010). Cohabitation enhances the avoidance response to heterospecific alarm cues in a freshwater snail. Anim. Behav..

[14] Aubier TG (2020). Positive density dependence acting on mortality can help maintain species-rich communities. Elife.

[15] Elias, M., Gompert, Z., Jiggins, C. & Willmott, K. Mutualistic interactions drive ecological niche convergence in a diverse butterfly community. *PLoS Biol.* **6**, e300 (2008). 10.1371/journal.pbio.0060300 PMC2592358 19055316

[16] Papageorgis C, C P (1975). Mimicry in Neotropical butterflies: Why are there so many different wing-coloration complexes in one place?. Am. Sci..

[17] Willmott KR, Mallet J (2004). Correlations between adult mimicry and larval host plants in ithomiine butterflies. Proc. R. Soc. Lond. B Biol. Sci..

[18] Gompert Z, Willmott K, Elias M (2011). Heterogeneity in predator micro-habitat use and the maintenance of Müllerian mimetic diversity. J. Theor. Biol..

[19] Estrada C, Jiggins CD (2008). Interspecific sexual attraction because of convergence in warning colouration: is there a conflict between natural and sexual selection in mimetic species?. J. Evol. Biol..

[20] Mérot C, Frérot B, Leppik E, Joron M (2015). Beyond magic traits: multimodal mating cues in Heliconius butterflies. Evolution.

[21] Welch JJ (2004). Accumulating Dobzhansky‐Muller incompatibilities: reconciling theory and data. Evolution.

[22] Aubier TG, Elias M, Llaurens V, Chazot N (2017). Mutualistic mimicry enhances species diversification through spatial segregation and extension of the ecological niche space. Evolution.

[23] González-Rojas M (2020). Chemical signals act as the main reproductive barrier between sister and mimetic Heliconius butterflies. Proc. R. Soc. Lond. B Biol. Sci..

[24] Blandin, P. *The Systematics of the Genus Morpho, Fabricius, 1807 (Lepidoptera Nymphalidae, Morphinae)* (Hillside Books, Canterbury, 2007).

[25] Llaurens V, Le Poul Y, Puissant A, Blandin P, Debat V (2020). Convergence in sympatry: evolution of blue-banded wing pattern in Morpho butterflies. J. Evol. Biol..

[26] Young AM (1971). Wing coloration and reflectance in *Morpho* butterflies as related to reproductive behavior and escape from avian predators. Oecologia.

[27] Páez E (2021). Hard to catch: experimental evidence supports evasive mimicry. Proc. R. Soc. Lond. B Biol. Sci..

[28] Grether GF, Peiman KS, Tobias JA, Robinson BW (2017). Causes and consequences of behavioral interference between species. Trends Ecol. Evol..

[29] Darragh K (2017). Male sex pheromone components in Heliconius butterflies released by the androconia affect female choice. PeerJ.

[30] Servedio MR, Noor MA (2003). The role of reinforcement in speciation: theory and data. Annu. Rev. Ecol. Evol. Syst..

[31] Smadja C, Ganem G (2008). Divergence of odorant signals within and between the two European subspecies of the house mouse. Behav. Ecol..

[32] Fraïsse, C. et al. DILS: demographic inferences with linked selection by using ABC. *Mol. Ecol. Resour.*10.1111/1755-0998.13323 (2021). 10.1111/1755-0998.13323 33448666

[33] Chazot N (2016). Morpho morphometrics: shared ancestry and selection drive the evolution of wing size and shape in *Morpho* butterflies. Evolution.

[34] Groot AT (2014). Circadian rhythms of sexual activities in moths: a review. Front. Ecol. Evol..

[35] Schöfl G, Heckel DG, Groot AT (2009). Time-shifted reproductive behaviours among fall armyworm (Noctuidae: *Spodoptera frugiperda*) host strains: evidence for differing modes of inheritance. J. Evol. Biol..

[36] Robinson SK, Terborgh J (1995). Interspecific aggression and habitat selection by Amazonian birds. J. Anim. Ecol..

[37] Brito J, Lizana M, Martínez-Freiría F, do Amaral JP (2010). Spatial and temporal segregation allows coexistence in a hybrid zone among two Mediterranean vipers (*Vipera aspis* and *V. latastei*). Amphib. Reptil..

[38] Schuett GW (2005). Sympatric rattlesnakes with contrasting mating systems show differences in seasonal patterns of plasma sex steroids. Anim. Behav..

[39] Taylor RS, Friesen VL (2017). The role of allochrony in speciation. Mol. Ecol..

[40] Gallard J-Y, Fernandez S (2017). Des postes territoriaux des Riodinides en Guyane: découverte de deux nouvelles espèces (Lepidoptera, Riodinidae). Bull. Soc. Entomol. Fr..

[41] Hirota T, Hamano K, Obara Y (2001). The influence of female post-emergence behavior on the time schedule of male mate-locating in Pieris rapae crucivora. Zool. Sci..

[42] Iwasa Y, Obara Y (1989). A game model for the daily activity schedule of the male butterfly. J. Insect Behav..

[43] Callaghan CJ (1982). A study of isolating mechanisms among Neotropical butterflies of the subfamily Riodininae. J. Res. Lepid..

[44] Freitas AV, Benson WW, Marini-Filho OJ, De Carvalho RM (1997). Territoriality by the dawn’s early light: the Neotropical owl butterfly *Caligo idomenaeus* (Nymphalidae: Brassolinae). J. Res. Lepid..

[45] Kemp DJ, Rutowski RL (2001). Spatial and temporal patterns of territorial mate locating behaviour in *Hypolimnas bolina* (L.) (Lepidoptera: Nymphalidae). J. Nat. Hist..

[46] Devries PJ, Austin GT, Martin NH (2008). Diel activity and reproductive isolation in a diverse assemblage of Neotropical skippers (Lepidoptera: Hesperiidae). Biol. J. Linn. Soc..

[47] Baird NA (2008). Rapid SNP discovery and genetic mapping using sequenced RAD markers. PLoS ONE.

[48] Catchen JM, Amores A, Hohenlohe P, Cresko W, Postlethwait JH (2011). Stacks: building and genotyping loci de novo from short-read sequences. G3 Genes Genomes Genet..

[49] Catchen J, Hohenlohe PA, Bassham S, Amores A, Cresko WA (2013). Stacks: an analysis tool set for population genomics. Mol. Ecol..

[50] Paris JR, Stevens JR, Catchen JM (2017). Lost in parameter space: a road map for stacks. Methods Ecol. Evol..

[51] Excoffier L, Dupanloup I, Huerta-Sánchez E, Sousa VC, Foll M (2013). Robust demographic inference from genomic and SNP data. PLoS Genet..

[52] Roux C (2016). Shedding light on the grey zone of speciation along a continuum of genomic divergence. PLoS Biol..

[53] Charlesworth B, Morgan M, Charlesworth D (1993). The effect of deleterious mutations on neutral molecular variation. Genetics.

[54] Cruickshank TE, Hahn MW (2014). Reanalysis suggests that genomic islands of speciation are due to reduced diversity, not reduced gene flow. Mol. Ecol..

[55] Roux C (2014). Can we continue to neglect genomic variation in introgression rates when inferring the history of speciation? A case study in a Mytilus hybrid zone. J. Evol. Biol..

[56] Gutenkunst, R., Hernandez, R., Williamson, S. & Bustamante, C. Diffusion approximations for demographic inference: DaDi. *Nat. Preced.*10.1038/npre.2010.4594.1 (2010).

[57] Jouganous J, Long W, Ragsdale AP, Gravel S (2017). Inferring the joint demographic history of multiple populations: beyond the diffusion approximation. Genetics.

[58] Pudlo P (2016). Reliable ABC model choice via random forests. Bioinformatics.

[59] Raynal L (2019). ABC random forests for Bayesian parameter inference. Bioinformatics.

[60] Baillargeon S, Rivest L-P (2007). Rcapture: loglinear models for capture-recapture in R. J. Stat. Softw.

[61] Garcia CR, Gallusser S, Lachaume L, Blandin P (2014). The ecology and life cycle of the Amazonian *Morpho cisseis* phanodemus Hewitson, 1869, with a comparative review of early stages in the genus *Morpho* (Lepidoptera: Nymphalidae: Morphinae). Trop. Lepid. Res..

[62] Hartley, R. & Zisserman, A. *Multiple View Geometry in Computer Vision* (Cambridge Univ. Press, Cambridge, 2003).

[63] Hedrick TL (2008). Software techniques for two-and three-dimensional kinematic measurements of biological and biomimetic systems. Bioinspir. Biomim..

[64] Theriault DH (2014). A protocol and calibration method for accurate multi-camera field videography. J. Exp. Biol..

[65] Muijres FT, Elzinga MJ, Melis JM, Dickinson MH (2014). Flies evade looming targets by executing rapid visually directed banked turns. Science.

[66] Lewis F, Butler A, Gilbert L (2011). A unified approach to model selection using the likelihood ratio test. Methods Ecol. Evol..

