# Supplementary material for: Convergent morphology and divergent phenology promote the coexistence of Morpho butterfly species
Source: Nat Commun. 2021 Dec 13;12:7248. doi: 10.1038/s41467-021-27549-1 (PMC8668891; doi:10.1038/s41467-021-27549-1)
Supplement: Supplementary file 1 — Supplementary Information [file 41467_2021_27549_MOESM1_ESM.pdf]

Supplementary Information

Convergent morphology and divergent phenology promote the coexistence of *Morpho* butterfly species

Authors list

Camille Le Roy[1,2,3*], Camille Roux[4], Elisabeth Authier[4], Hugues Parrinello[5], Héloïse Bastide[6], Vincent Debat[1] & Violaine Llaurens[1]

Affiliations

[1] Institut de Systématique, Evolution, Biodiversité (ISYEB), Muséum National d'Histoire Naturelle, CNRS, Sorbonne Université, EPHE, Université des Antilles, CP50, 75005 Paris, France.
[2] Université Paris Descartes, Sorbonne Paris Cité, 12 rue de l'École de Médecine, 75006 Paris, France.
[3]Department of Experimental Zoology, Wageningen University, 6709 PG Wageningen, the Netherlands.
[4] Univ. Lille, CNRS, UMR 8198 - Evo-Eco-Paleo, F-59000 Lille, France.
[5] MGX-Montpellier GenomiX, Univ. Montpellier, CNRS, INSERM, F34094 Montpellier France.
[6] Université Paris-Saclay, CNRS, IRD, UMR Évolution, Génomes, Comportement et Écologie, 91198, Gif-sur-Yvette, France.

These authors contributed equally: Vincent Debat, Violaine Llaurens.

*corresponding author: Camille Le Roy

Email: leroy.camille7@gmail.com

Supplementary Figures

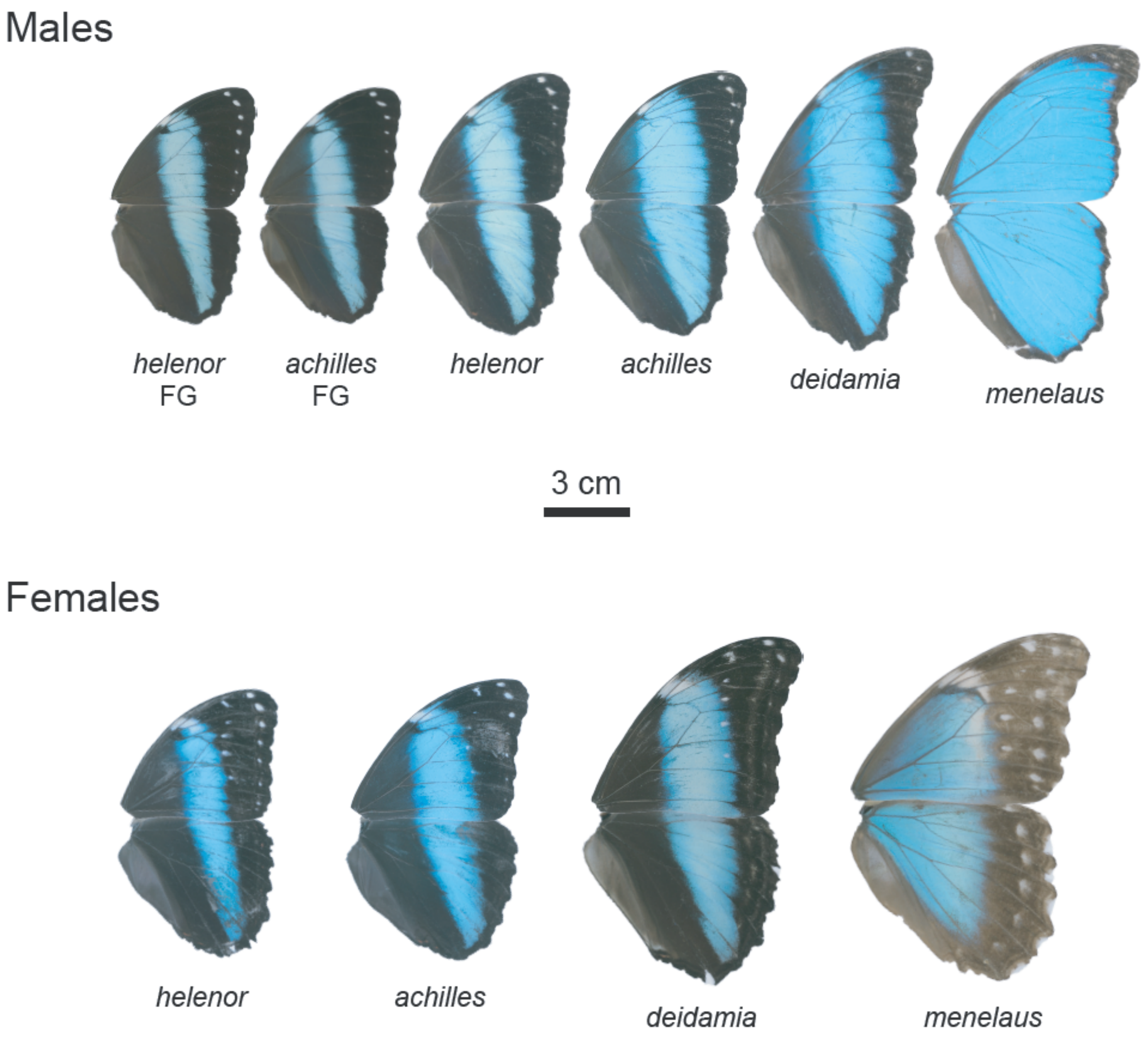

Supplementary Figure 1. Wings of *Morpho* butterflies used for the dummy experiment. 'FG' indicates exotic dummies from French Guiana. All other dummies were build with wings from Peruvian individuals. Wings are shown at their relative size.

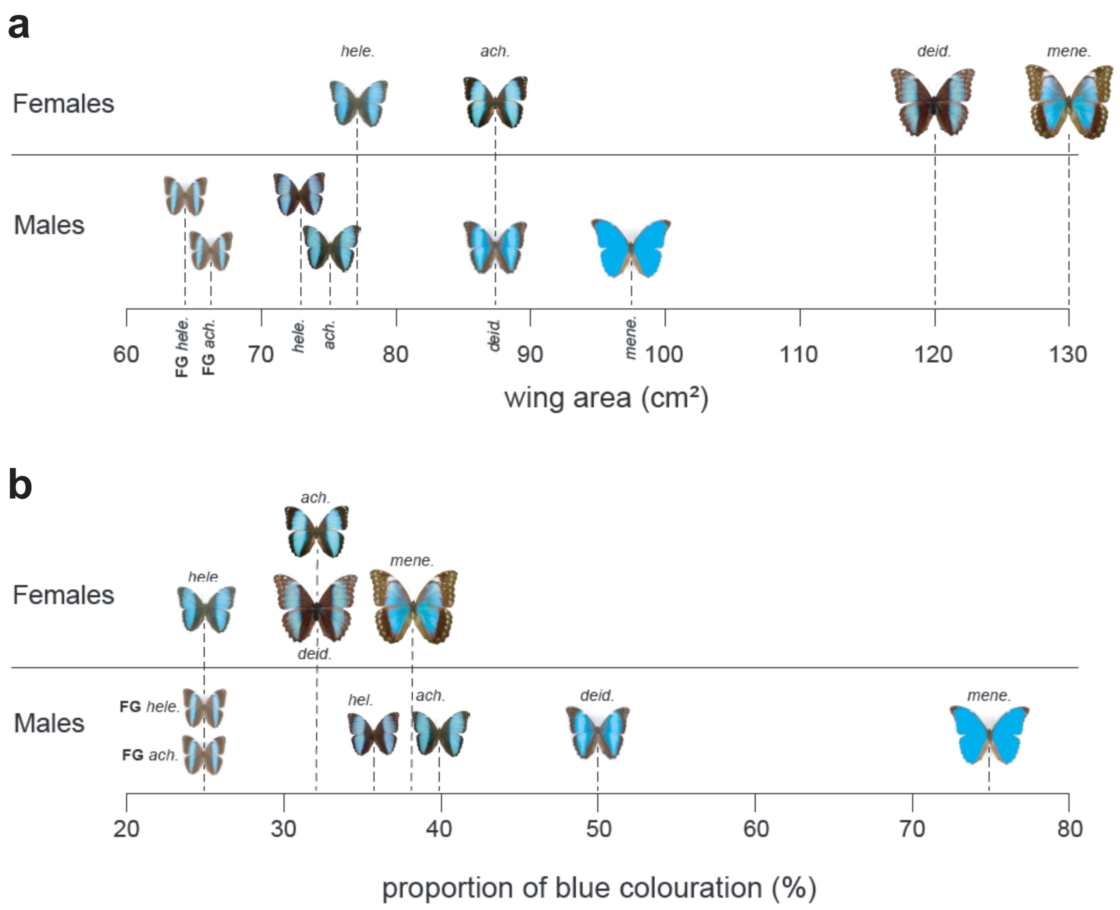

Supplementary Figure 2. (**a**) Variation in wing area and (**b**) in proportion of blue colouration among the tested dummy butterflies. 'FG' indicates exotic dummies from French Guiana. All other dummies were build with wings from Peruvian individuals. *hele: Morpho helenor; ach: Morpho achilles; deid: Morpho deidamia; mene: Morpho menelaus.*

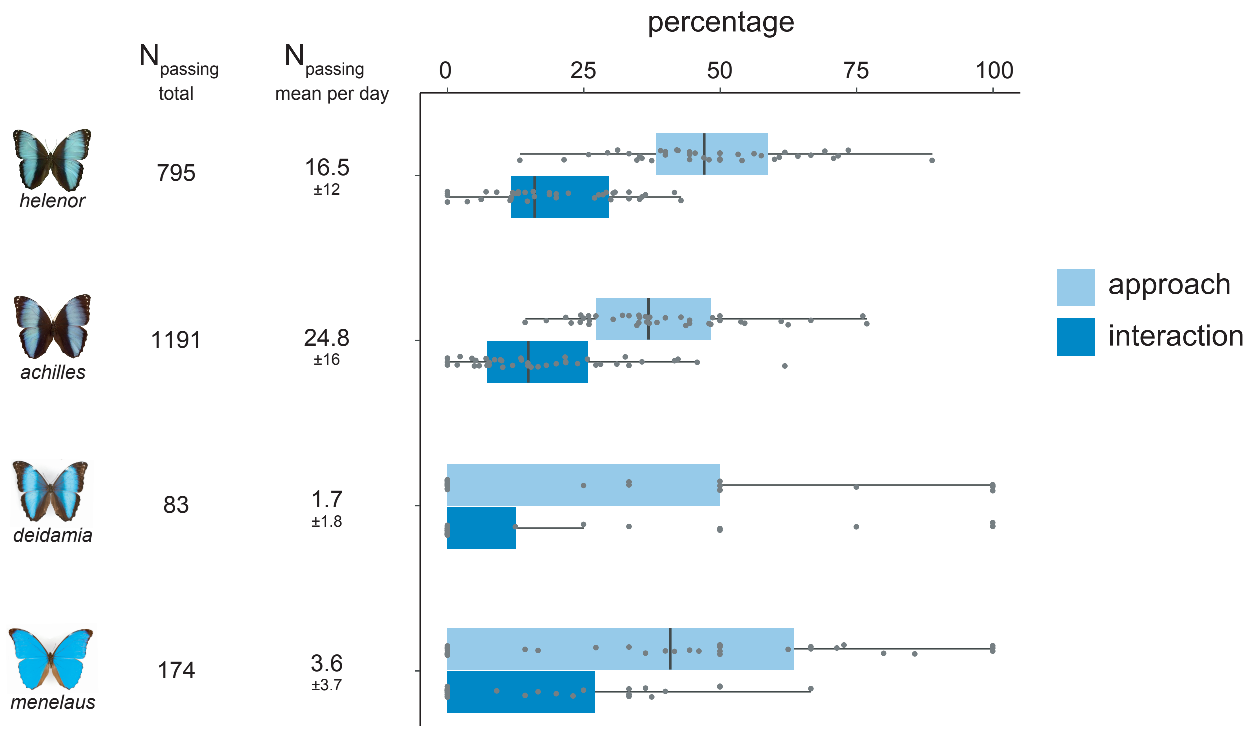

Supplementary Figure 3. Percentage of approach and interaction with the dummy butterfly (all dummy identity and sex confounded) among sympatric *Morpho* species. Percentage were computed over the number of passing individuals along the river. Total number of passing *Morpho* (in 40 day of experiment) and mean per day is indicated on the left. Each point on the boxplot is a different day of experiment. Boxplots show the median and inter-quartile range (IQR), while whiskers depict the data range (75th and 25th ±1.5*IQR, respectively). Source data are provided as a Source Data file.

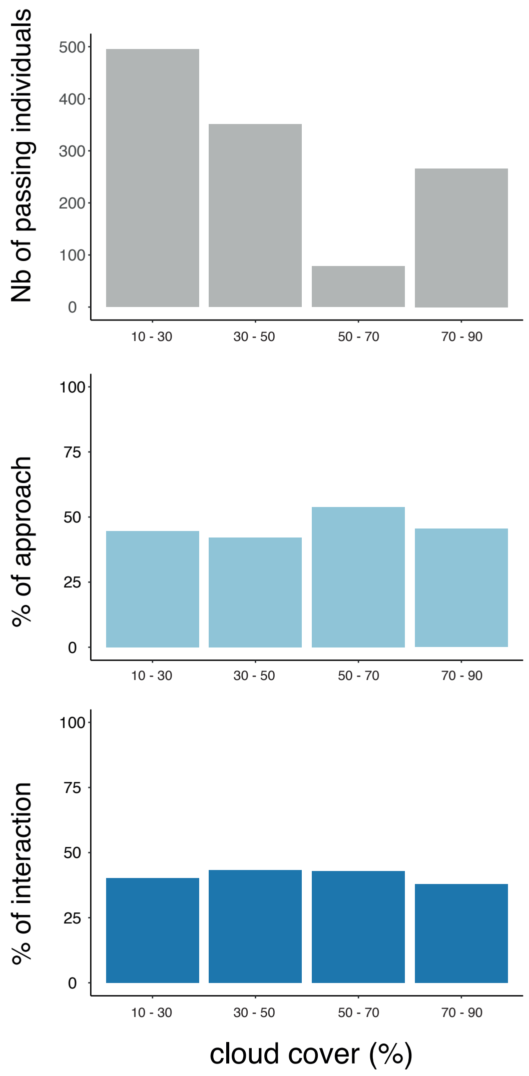

Supplementary Figure 4. Effect of cloud cover on the number of passing butterflies (top), the percentage of approach (middle) and of interaction (bottom) with the dummy. Source data are provided as a Source Data file.

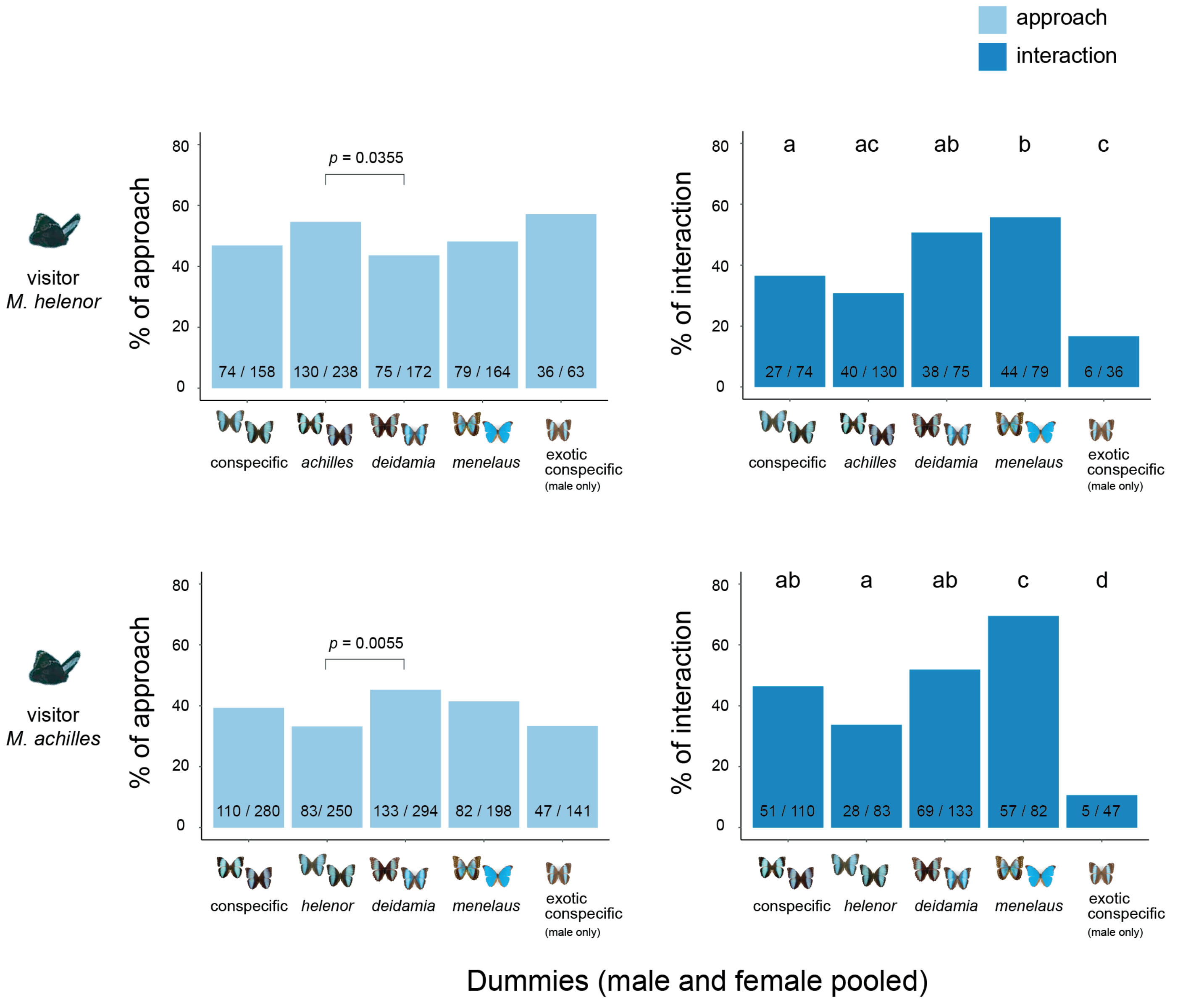

Supplementary Figure 5. Frequency of approach (left column) and interaction (right column) with conspecific and congener dummies in two sister *Morpho* species. Female and male dummies are pooled together, excepted for the exotic dummies where only males were tested. Raw data « nb of approaches / nb of passing» are indicated on bars of the left column. « nb of interactions / nb of approaches» are indicated on bars of the right column. Proportions were compared using Fisher Exact probability tests. Only significant differences are shown ($p < 0.05$). On the right column, the letters a, b, c, and d indicate statistically significant difference at $p < 0.05$. Bars with no common letters are significantly different ($p < 0.05$). Source data are provided as a Source Data file.

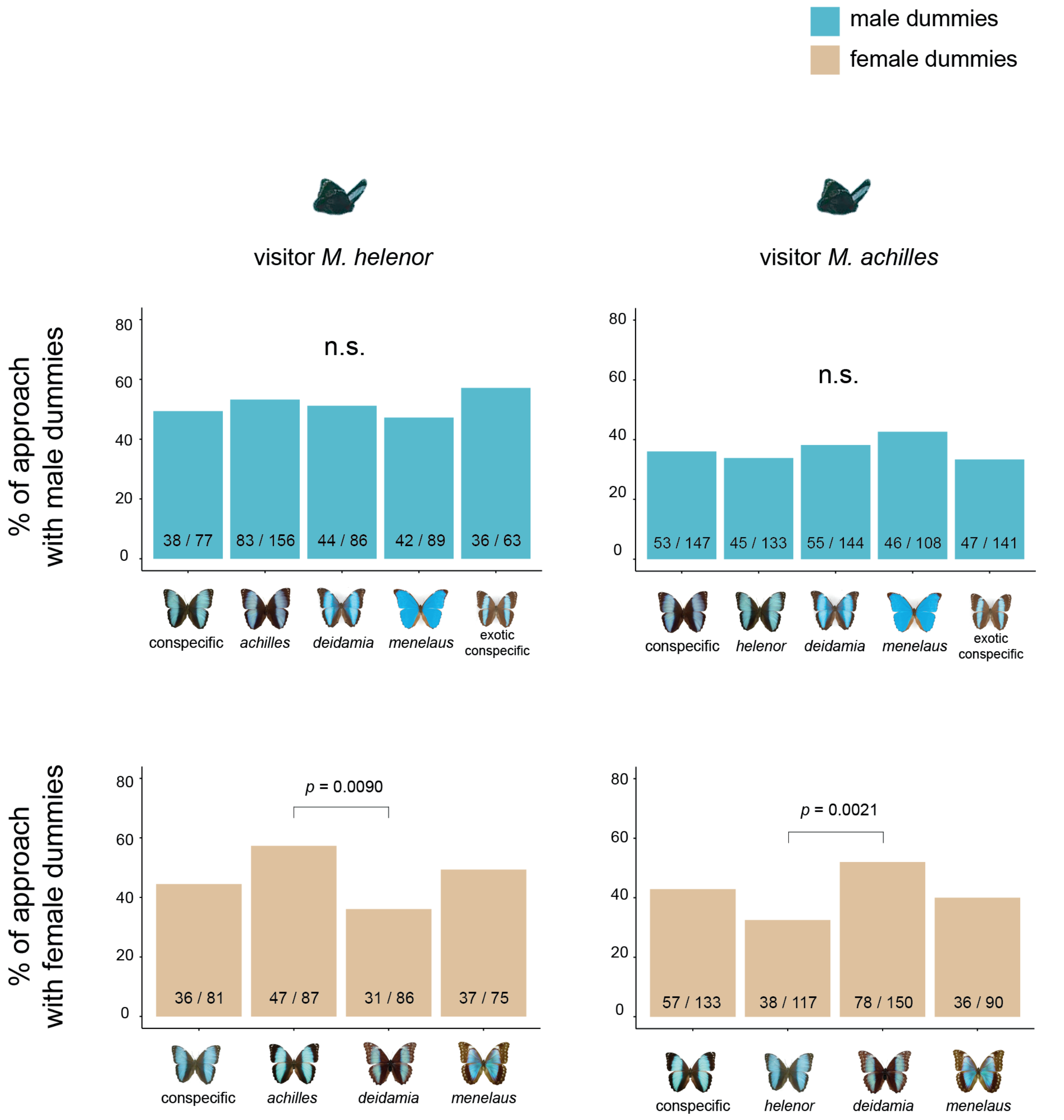

Supplementary Figure 6. Approach frequency with conspecific and congener dummies in two sister *Morpho* species. Raw data « nb of approaches / nb of passing » are indicated on each bar. Proportions were compared using Fisher Exact probability tests. Only significant differences are shown ($p < 0.05$). ns: not significant. Source data are provided as a Source Data file.

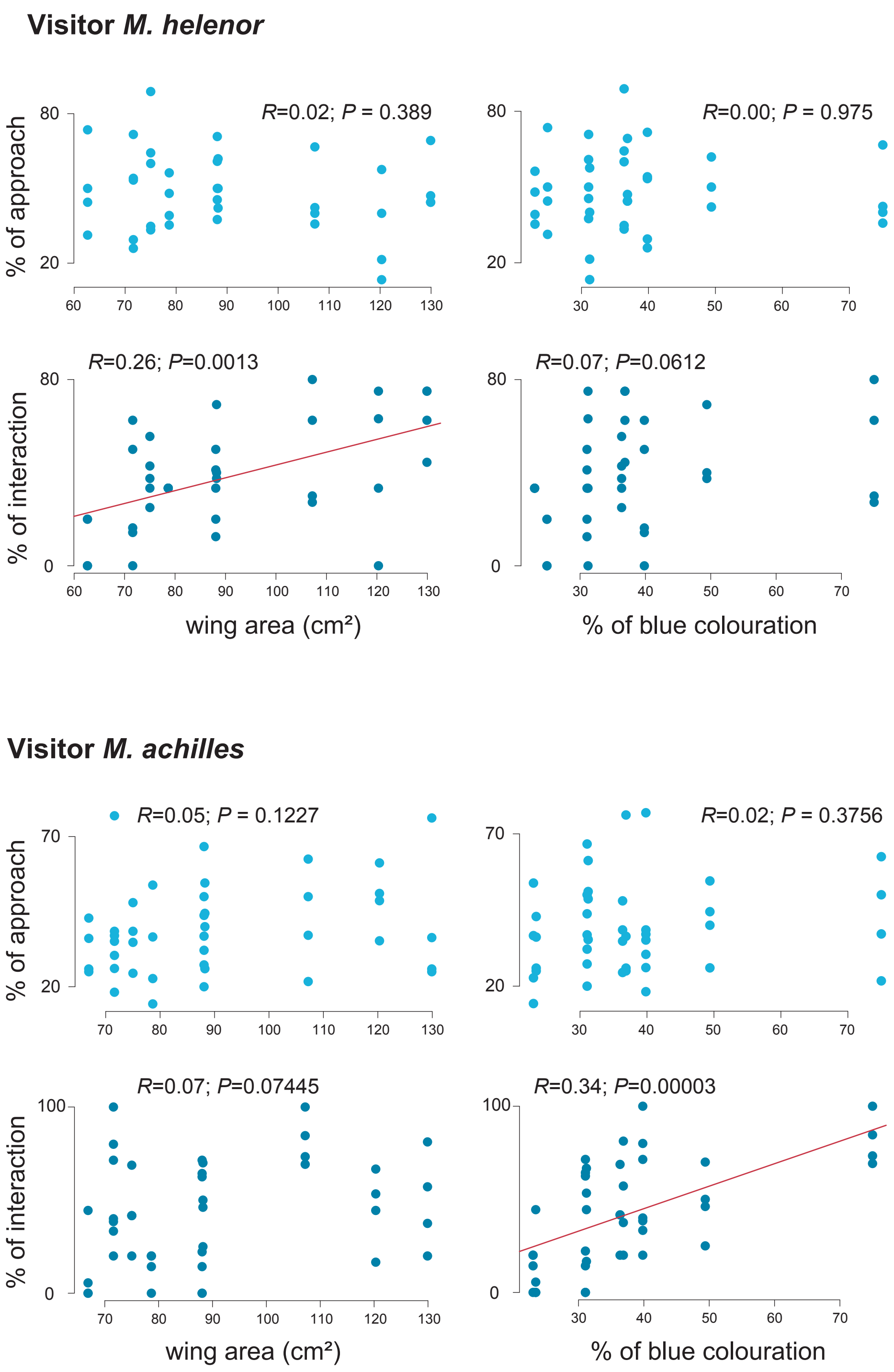

Supplementary Figure 7. The percentage of approach (top row, light blue) and the percentage of interaction (bottom row, dark blue) are showed in relation to the area and the proportion of blue colouration on the dummy wings. Top panel: visitor *M. helenor*. Bottom panel visitor *M. achilles*. Relationships were tested using linear regressions. Regression lines are plotted in cases of significant relationships. Each point is a different day of experiment. Source data are provided as a Source Data file.

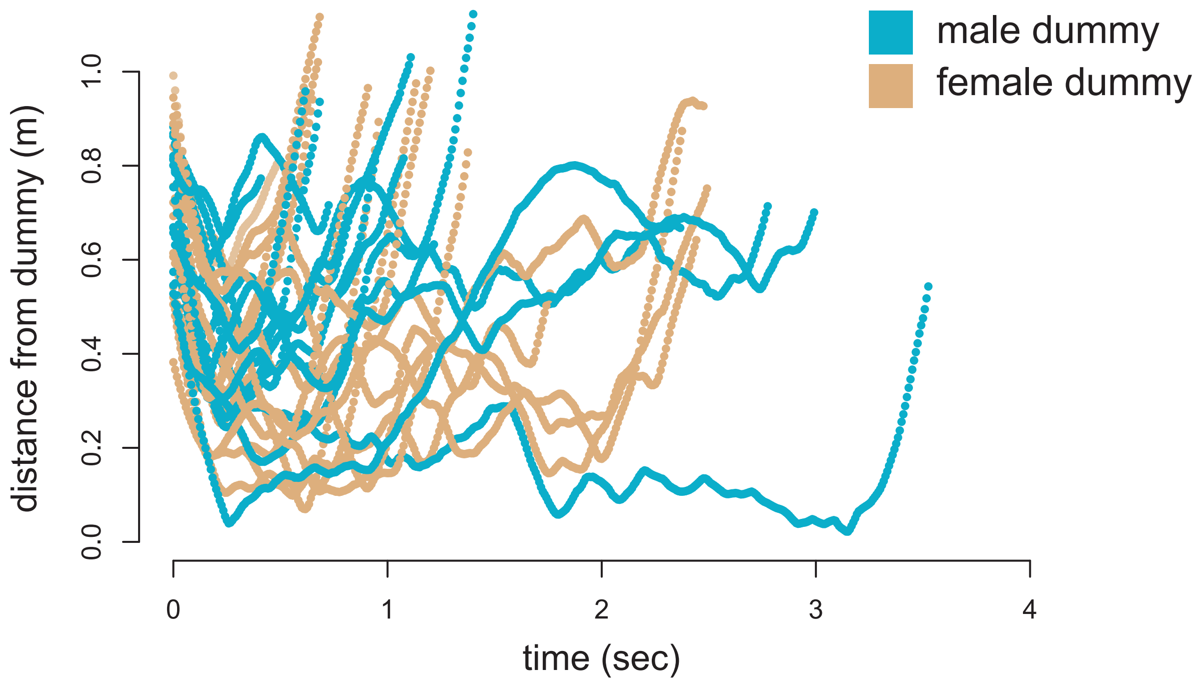

Supplementary Figure 8. Variation in distance between visitor male *Morpho* achilles and the dummy during the interaction. Blue and yellow colors indicate male and female conspecific dummy, respectively. Source data are provided as a Source Data file.

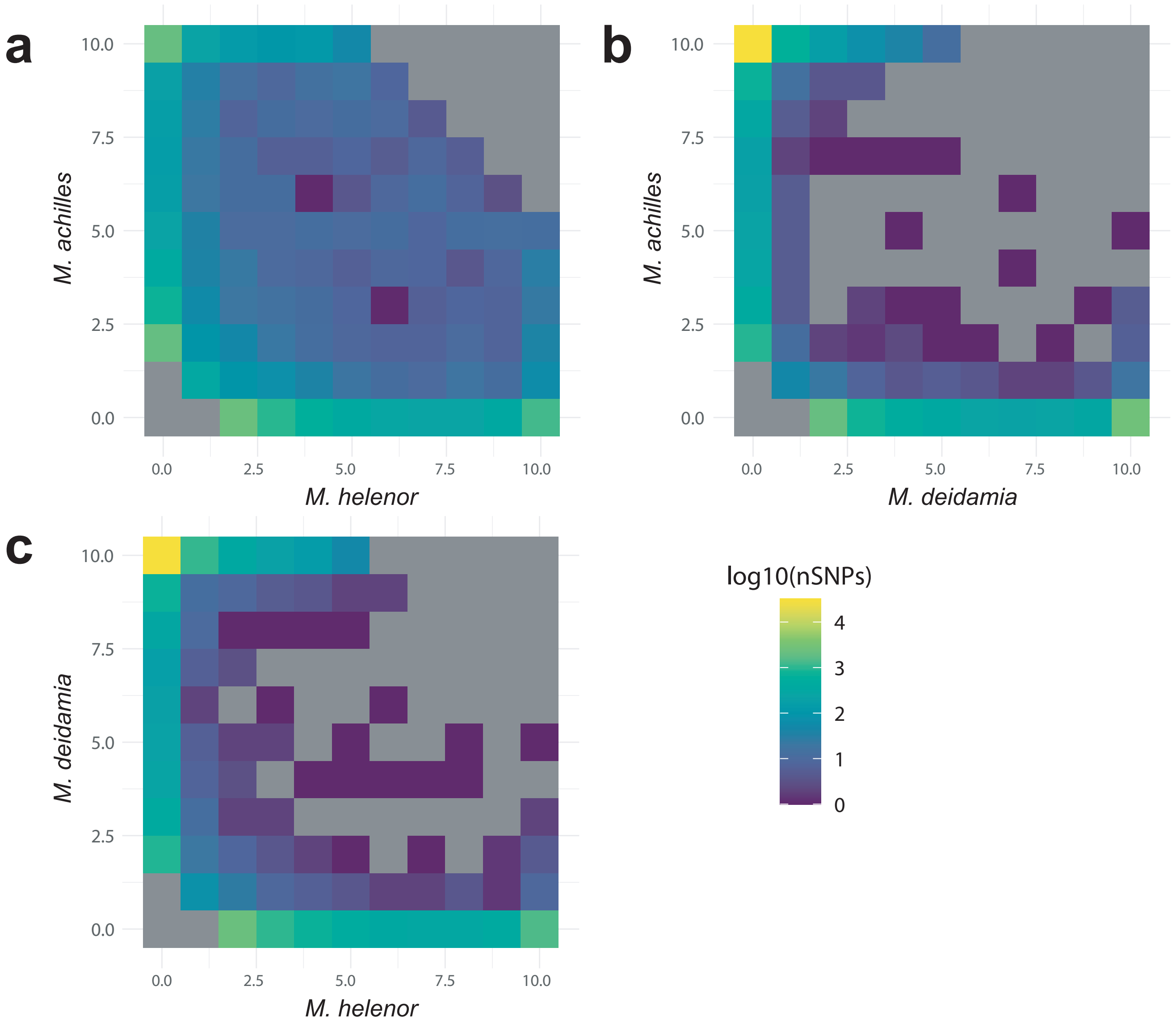

Supplementary Figure 9. Joint spectra of the allelic frequencies between *M. helenor*, *M. achilles* and *M. deidamia*. Folded spectrum based on the frequency of the minority allele for each polymorphic position when the three species are aligned. The intraspecific singletons have been removed from the graphical representation to avoid upscaling. Spectra are represented on the log10 scale for three different pairs **a**) *M. helenor – M. achilles*; **b**) *M. deidamia – M. achilles*; **c**) *M. helenor – M. deidamia*. Source data are provided as a Source Data file.

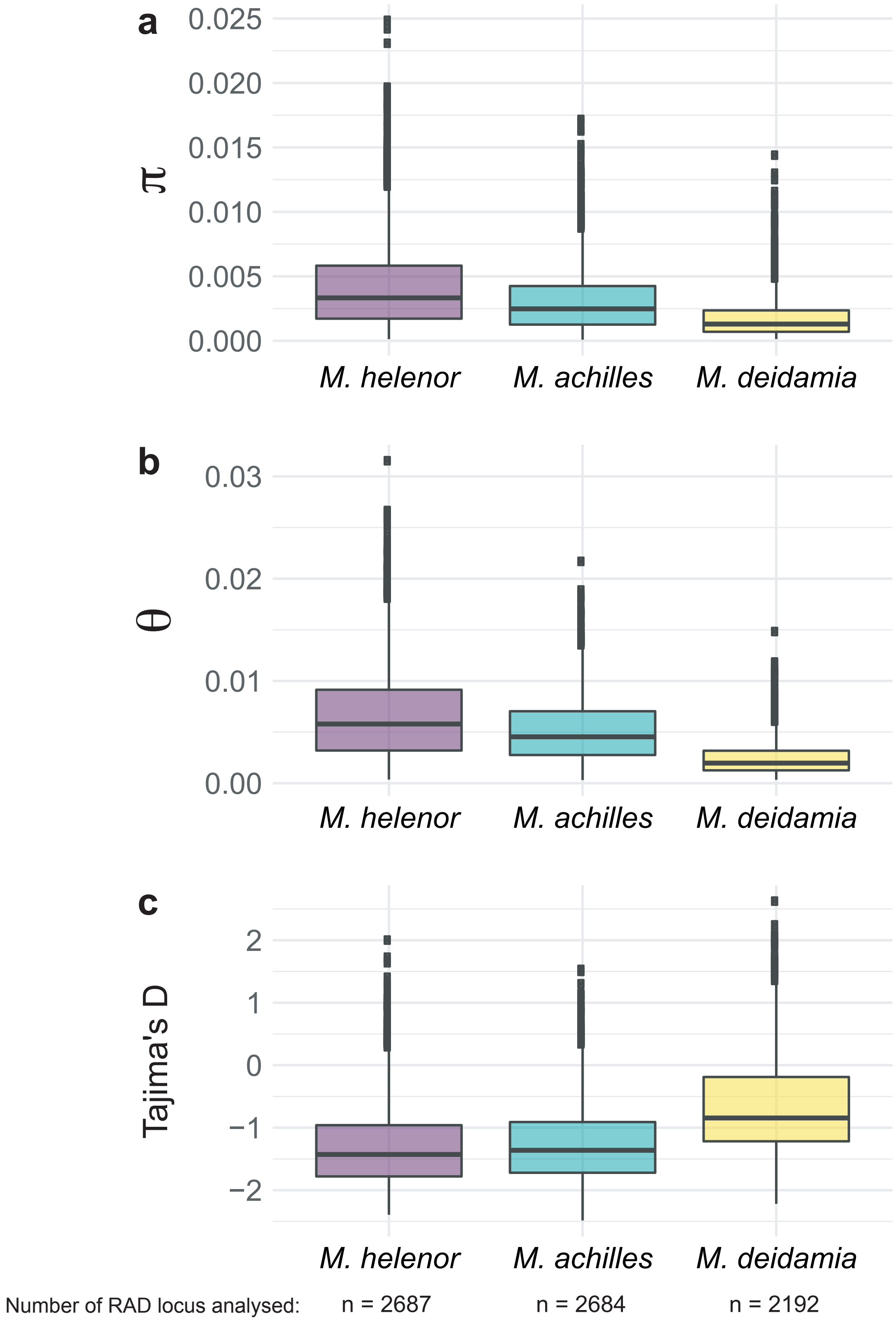

Supplementary Figure 10. Patterns of within-species molecular diversity**.**
a) π (Tajima, 1983); **b**) θ (Watterson, 1975); **c**) Taijma's D (Tajima, 1989).
Boxplots show the median and inter-quartile range (IQR), while whiskers depict the data range (75th and 25th ±1.5*IQR, respectively). Source data are provided as a Source Data file.

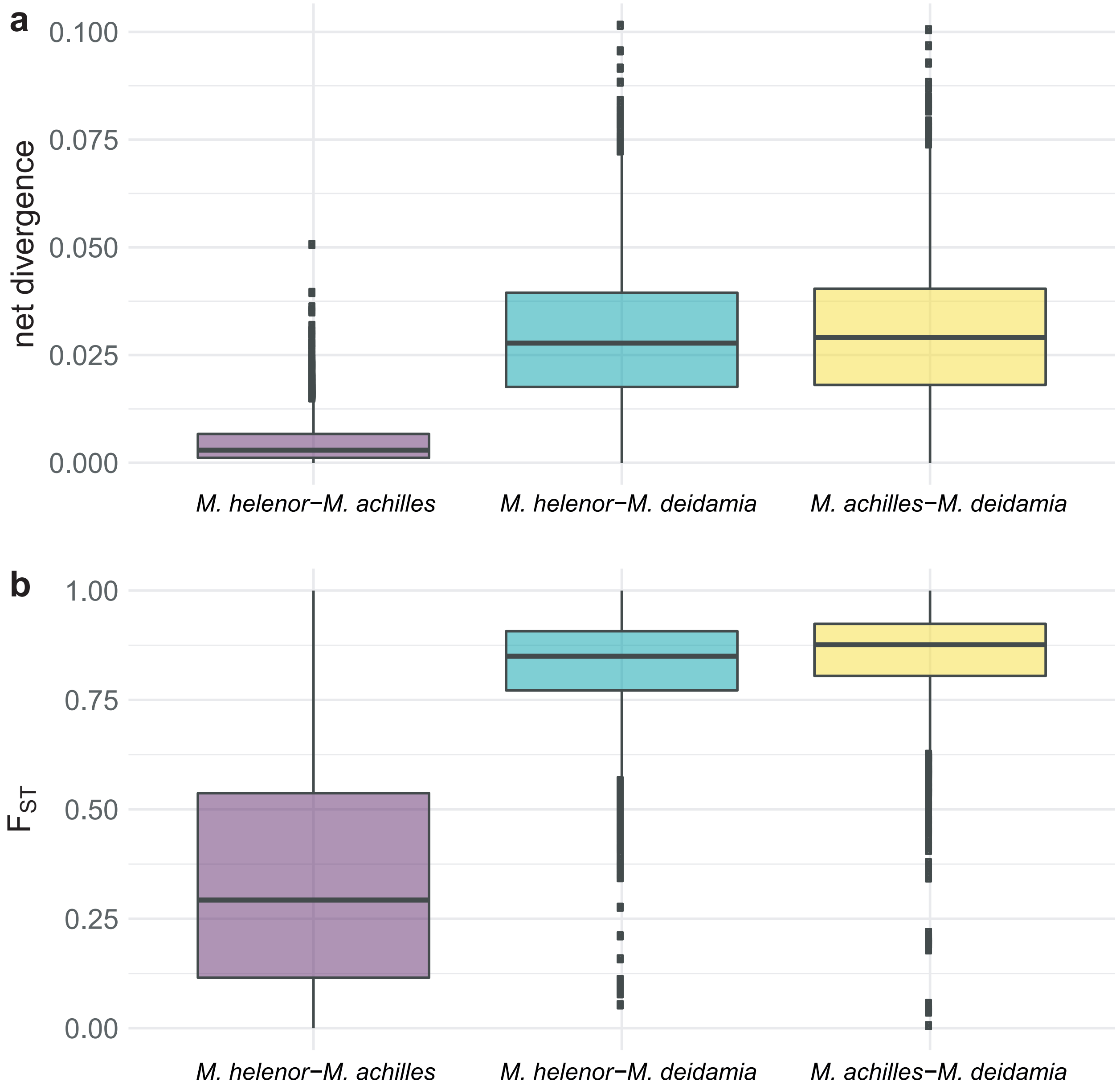

Number of RAD locus analysed: N = 2486 N = 2734 N = 2732

Supplementary Figure 11. Patterns of between-species divergence and differentiation**. a**) net divergence measured Da (Nei, 1987). **b**) Fst computed as (1 - πs) / πT where πs is the average pairwise nucleotide diversity within population and πT is the total pairwise nucleotide diversity of the pooled sample across populations. Boxplots show the median and inter-quartile range (IQR), while whiskers depict the data range (75th and 25th ±1.5*IQR, respectively). Source data are provided as a Source Data file.

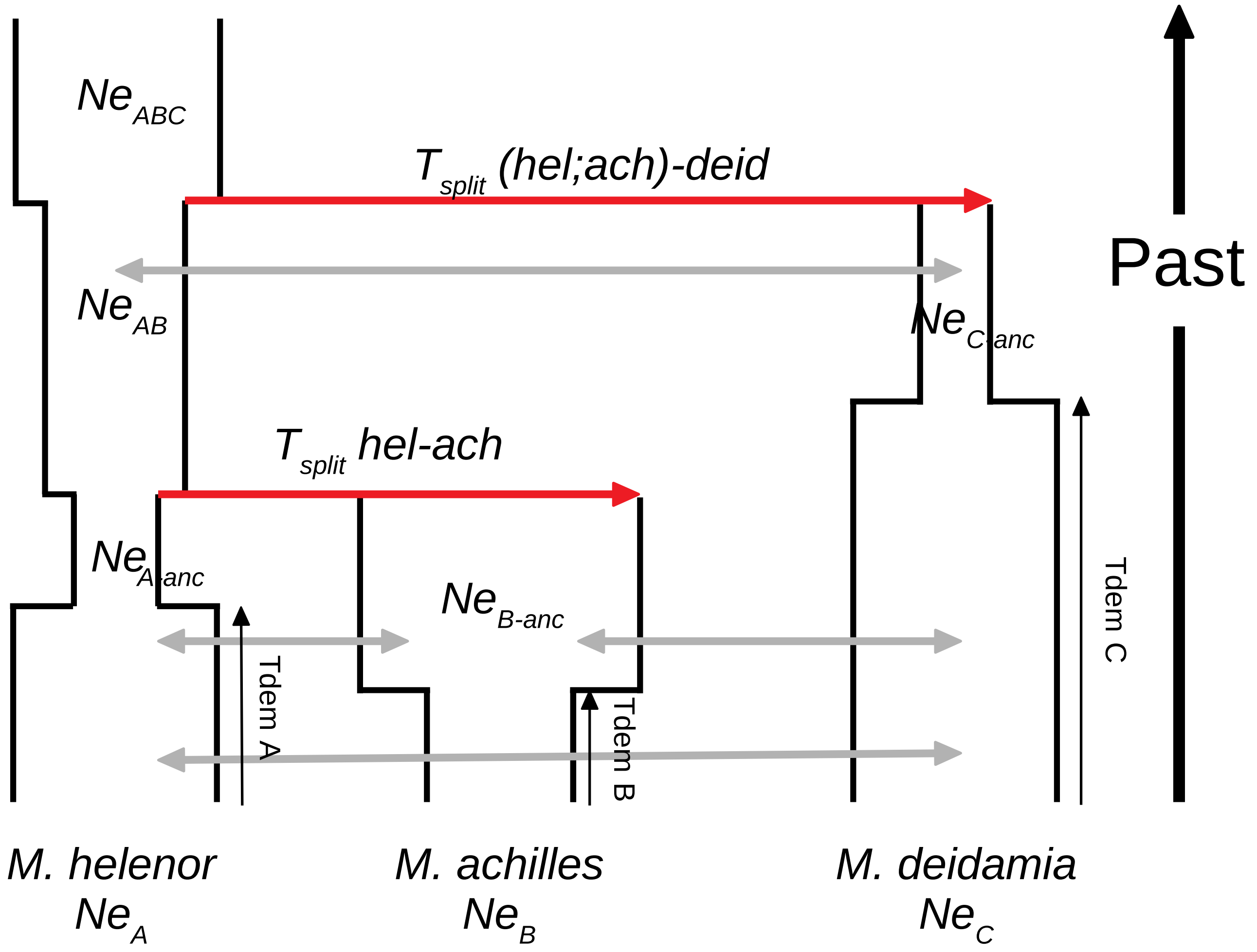

Supplementary Figure 12. General model of 3 demes speciation with gene flow. This model describes two successive splitting events from the ancestor (size NeABC) to the three sampled species (sizes NeA; NeB; NeC). Each split event is followed by the drawing of a new population size (after the first split: NeAB; NeC–anc. After the second split: NeAC–anc; NeBC–anc). Populations may also undergo a demographic change in size at some point in their recent history, at times Tdem–A, Tdem–B and Tdem–C. This demographic change consists of the sampling of a new population size that may be larger or smaller than the size of their ancestor. Two migration relationships are considered: 1) migration between C and (A;B). 2) migration between A and B. Concerning the first migration relationship, 4 scenarios are explored: migration only between A and C; only between B and C; between A-C and B-C; no migration at all. Concerning the second migration relationship, 2 scenarios are explored: ancient migration (restricted to the first generations after a split) and secondary contact (isolation after a split then contact with gene flow). Ages of migration changes are randomly drawn between zero and the age of the split leading to the concerned lineages.

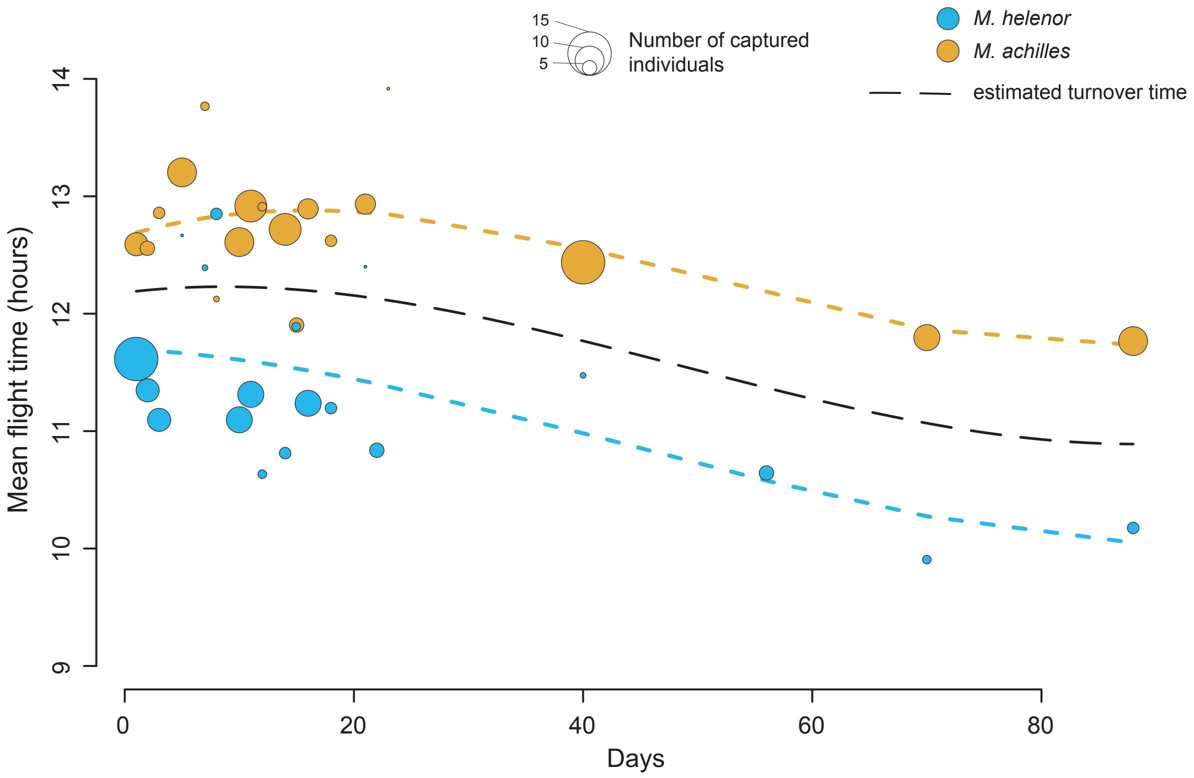

Supplementary Figure 13. Plot of the mean flight time of the two sister species *M. helenor* and *M. achilles* over a ~three-month period. Capture sessions were performed on consecutive days during the first 20 day of experiment. We then performed one day of capture every 2 weeks during 2 months in parallel to the dummy experiment to verify that temporal activity was stable over time. Colored dashed lines are the estimated mean flight time for *M. helenor* and *M. achilles* (blue and orange respectively). Because turnover time slightly change over the duration of the experiment, we estimated the identify of the flying *Morpho* during the dummy experiment based on the hours of the day and the turnover time estimated for this day (black dashed line). Source data are provided as a Source Data file.

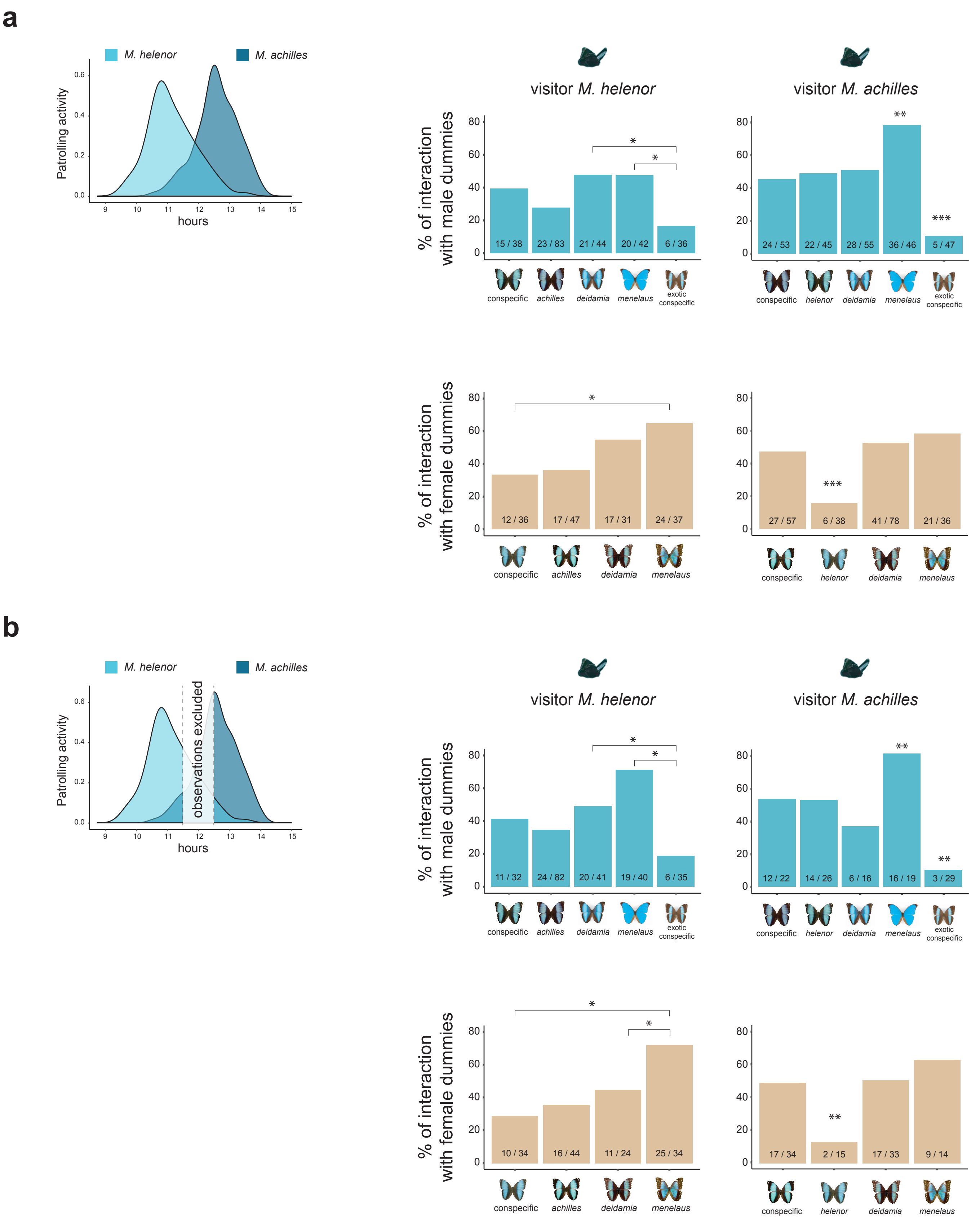

Supplementary Figure 14. Because *M. helenor* and *M. achilles* cannot be distinguished when flying, we used time the day as a predictor of species identity (patrolling activity of the two species is shown in top left corner). Patrolling time of the two species nonetheless overlap, preventing to confidently predict species identity during the overlapping time window. We thus ran the analysis while removing the observations made between 11:30 am and 12:30 pm to verify whether the results observed in our main analysis (panel **a**) were consistent with the results obtained using the subsample (panel **b**). Proportions were compared using Fisher Exact probability tests. Stars indicates statistically significant difference (**p* < 0.05; ***p* < 0.01; ****p* < 0.001). Source data are provided as a Source Data file.

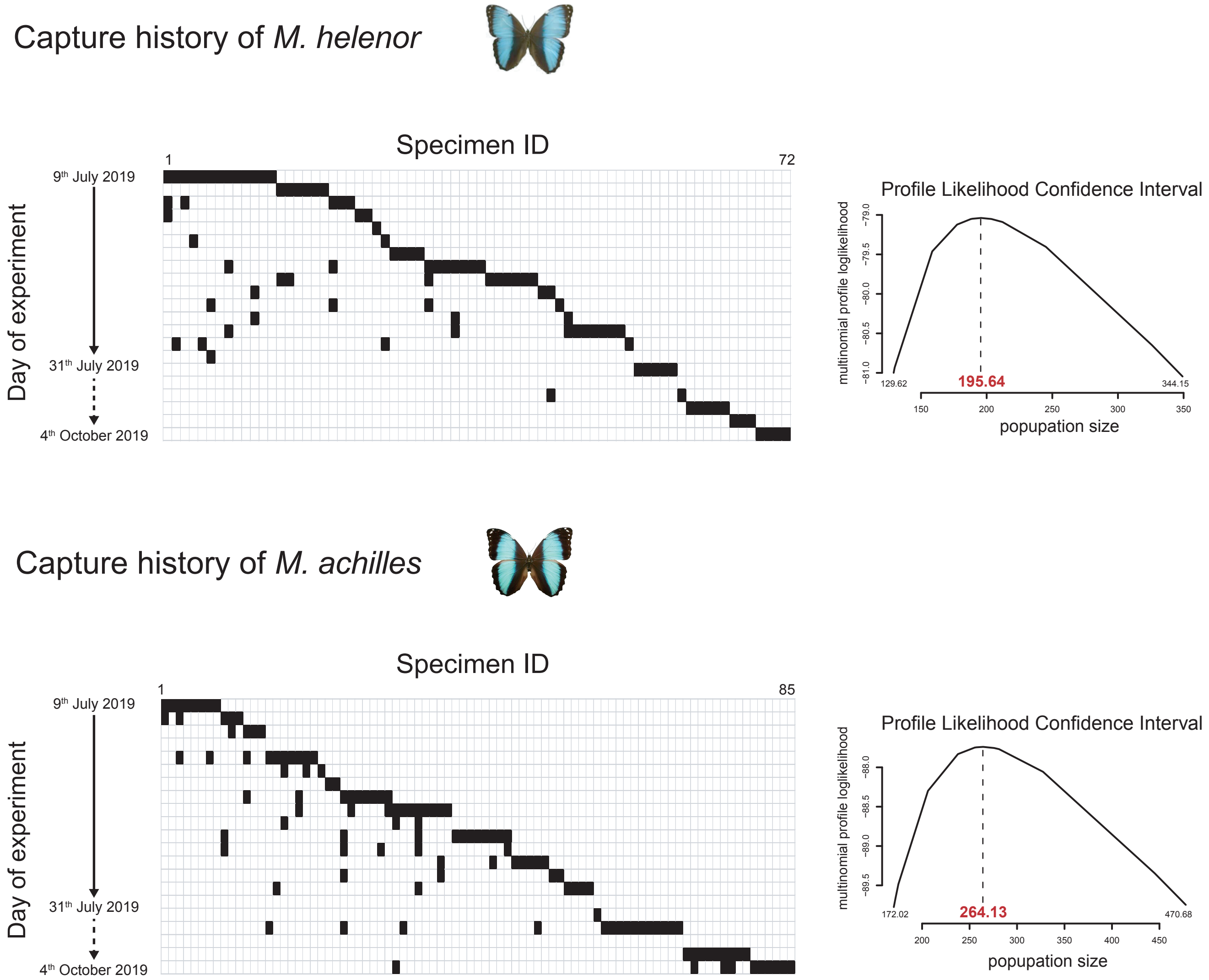

Supplementary Figure 15. Estimating population size from mark-recapture data. Capture history is shown for the two mimetic sister species *M. helenor* (top) and *M. achilles* (bottom). It gives the capture status on each day of experiment: caught (black boxes) or uncaught (white boxes). Days of experiment along the continuous arrow were nearly consecutive, while those along the dashed arrow were performed every 2 weeks. Based on capture-recapture histories, we estimated individual abundance for each species using a loglinear model implemented in the R package Rcapture (Baillargeon & Rivest 2007), assuming constant population size throughout the experiment. The likelihood confidence interval of the population sizes is shown on the right column. Source data are provided as a Source Data file.

Supplementary Tables

Supplementary Table 1. Number of captures and recaptures per *Morpho* species. Source data are provided as a Source Data file.

Species	Total number of capture	Total number of recapture	Mean % of recapture per day
M. helenor	92	24	23.9
M. achilles	121	36	29.7
M. deidamia	48	11	22.9
M. menelaus	34	5	14.7

Supplementary Table 2. Count data of observations between patroling male (columns) and dummies (rows).

		Visitor *M. helenor* ♂			Visitor *M. achilles* ♂			Visitor *M. deidamia* ♂			Visitor *M. menelaus* ♂		
		passing	approach	interaction	passing	approach	interaction	passing	approach	interaction	passing	approach	interaction
Dummy *M. helenor*	♂	77	38	15	133	45	22	11	2	1	22	12	6
Dummy *M. helenor*	♀	81	36	12	117	38	6	8	2	1	18	7	0
Dummy *M. achilles*	♂	156	83	23	147	53	24	12	1	1	29	8	4
Dummy *M. achilles*	♀	82	47	17	133	57	27	11	3	1	37	14	4
Dummy *M. deidamia*	♂	86	44	21	144	55	28	11	2	2	15	9	4
Dummy *M. deidamia*	♀	86	31	17	150	78	41	6	0	0	21	5	2
Dummy *M. menelaus*	♂	89	42	20	108	46	36	9	6	6	14	10	4
Dummy *M. menelaus*	♀	75	37	24	90	36	21	9	4	1	12	8	4
Dummy French Guiana *hel*	♂	63	36	6		not tested			not tested			not tested	
Dummy French Guiana *ach*	♂		not tested		141	47	5						

Supplementary Table 3. **Effect of dummy identity (both sex pooled) on the number of approaches and interactions**. The effect of dummy characteristics and of cloud cover on the number of approaches and interactions was tested using logistic regression models. Below are reported the results of likelihood ratio tests comparing models in order to test the global effect of each variable on the number of approaches and interactions. Source data are provided as a Source Data file.

Visitor *M. helenor*

	Dummy identity			Dummy sex			Dummy wing area			Dummy blue proportion			Cloud cover		
	D	*df*	*P*	*D*	*df*	*P*	*D*	*df*	*P*	*D*	*df*	*P*	*D*	*df*	*P*
Approach	12.6	4	**0.01**	0.8	1	0.35	0.20	1	0.65	0.72	1	0.39	0.42	1	0.51
Interaction	25.6	4	**0.00**	0.6	1	0.43	3.3	1	0.06	1.0	1	0.30	0.62	1	0.42

Visitor *M. achilles*

	Dummy identity			Dummy sex			Dummy wing area			Dummy blue proportion			Cloud cover		
	D	*df*	*P*	*D*	*df*	*P*	*D*	*df*	*P*	*D*	*df*	*P*	*D*	*df*	*P*
Approach	11.4	4	**0.02**	3.5	1	0.06	1.7	1	0.18	4.8	1	**0.02**	0.78	1	0.37
Interaction	60.2	4	**0.00**	2.4	1	0.12	1.1	1	0.28	6.5	1	**0.01**	0.99	1	0.31

Supplementary Table 4. **Effect of dummy identity (sex separated) on the number of approaches and interactions**. The effect of dummy identity and cloud cover on the number of approach and interaction was tested using logistic regression models. Below are reported the results of likelihood ratio tests comparing models in order to test the global effect of each variable on the number of approaches and interactions. Source data are provided as a Source Data file.

Visitor *M. helenor*

	Male dummies						Female dummies					
	Dummy identity			Cloud cover			Dummy identity			Cloud cover		
	D	*df*	*P*	*D*	*df*	*P*	*D*	*df*	*P*	*D*	*df*	*P*
Approach	4.7	4	0.31	0.29	1	0.59	7.9	3	0.50	0.32	1	0.56
Interaction	12.8	4	**0.01**	0.02	1	0.88	12.1	3	**0.00**	1.97	1	0.16

Visitor *M. achilles*

	Male dummies						Female dummies					
	Dummy identity			Cloud cover			Dummy identity			Cloud cover		
	D	*df*	*P*	*D*	*df*	*P*	*D*	*df*	*P*	*D*	*df*	*P*
Approach	2.7	4	0.59	0.27	1	0.60	14.9	3	**0.00**	0.58	1	0.44
Interaction	55.6	4	**0.00**	0.11	1	0.73	19.8	3	**0.00**	1.94	1	0.16

Supplementary Table 5. **Effect of dummy identity (both sex pooled) on the number of approaches and interactions tested on the subdataset excluding uncertainties on visitor identity**.
The effect of dummy characteristics and of cloud cover on the number of approaches and interactions was tested using logistic regression models. Below are reported the results of likelihood ratio tests comparing models in order to test the global effect of each variable on the number of approaches and interactions. Source data are provided as a Source Data file.

Visitor *M. helenor*

	Dummy identity			Dummy sex			Dummy wing area			Dummy blue proportion			Cloud cover		
	D	*df*	*P*	*D*	*df*	*P*	*D*	*df*	*P*	*D*	*df*	*P*	*D*	*df*	*P*
Approach	15.5	4	**0.00**	2.2	1	0.13	1.2	1	0.26	0.0	1	0.97	0.39	1	0.52
Interaction	26.8	4	**0.00**	0.9	1	0.33	1.1	1	0.28	3.1	1	0.07	0.46	1	0.49

Visitor *M. achilles*

	Dummy identity			Dummy sex			Dummy wing area			Dummy blue proportion			Cloud cover		
	D	*df*	*P*	*D*	*df*	*P*	*D*	*df*	*P*	*D*	*df*	*P*	*D*	*df*	*P*
Approach	6.0	4	0.19	4.4	1	**0.03**	0.7	1	0.39	0.7	1	0.39	0.83	1	0.36
Interaction	26.8	4	**0.00**	0.9	1	0.33	1.1	1	0.28	3.1	1	0.07	0.46	1	0.49

Supplementary Table 6. Effect of dummy identity (sex separated) on the number of approaches and interactions tested on the subdataset excluding uncertainties on visitor identity.
The effect of dummy identity and cloud cover on the number of approach and interaction was tested using logistic regression models. Below are reported the results of likelihood ratio tests comparing models in order to test the global effect of each variable on the number of approaches and interactions. Source data are provided as a Source Data file.

Visitor *M. helenor*

	Male dummies						Female dummies					
	Dummy identity			Cloud cover			Dummy identity			Cloud cover		
	D	*df*	*P*	*D*	*df*	*P*	*D*	*df*	*P*	*D*	*df*	*P*
Approach	8.5	4	0.07	0.42	1	0.51	5.23	3	0.15	0.25	1	0.61
Interaction	12.0	4	**0.01**	0.15	1	0.69	16.3	3	**0.00**	0.65	1	0.41

Visitor *M. achilles*

	Male dummies						Female dummies					
	Dummy identity			Cloud cover			Dummy identity			Cloud cover		
	D	*df*	*P*	*D*	*df*	*P*	*D*	*df*	*P*	*D*	*df*	*P*
Approach	3.5	4	0.47	0.12	1	0.72	6.5	3	0.08	0.97	1	0.32
Interaction	32.7	4	**0.00**	0.61	1	0.43	9.9	3	**0.01**	0.00	1	0.98

Supplementary Table 7. **Effect of wing area and blue proportion of the dummies on the number of approaches and interactions**. The effect of dummy wing area and proportion of blue colouration on the number of approaches and interactions was tested using logistic regression models. Below are reported the results of likelihood ratio tests comparing model in order to test the global effect of each variable on the number of approaches and interactions. Source data are provided as a Source Data file.

Visitor (all species)

	Dummy wing area			Dummy blue proportion			Cloud cover		
	D	*df*	*P*	*D*	*df*	*P*	*D*	*df*	*P*
Approach	2.60	1	0.10	0.39	1	0.52	0.92	46	0.33
Interaction	48.5	1	**0.00**	12.9	1	**0.00**	0.00	46	0.95

Visitor *M. helenor*

	Dummy wing area			Dummy blue proportion			Cloud cover		
	D	*df*	*P*	*D*	*df*	*P*	*D*	*df*	*P*
Approach	1.7	1	0.18	0.98	1	0.33	2.7	36	0.10
Interaction	25.1	1	**0.00**	0.12	1	0.72	0.02	36	0.87

Visitor *M. achilles*

	Dummy wing area			Dummy blue proportion			Cloud cover		
	D	*df*	*P*	*D*	*df*	*P*	*D*	*df*	*P*
Approach	11.0	1	**0.00**	0.0	1	0.82	0.55	40	0.45
Interaction	28.3	1	**0.00**	30.1	1	**0.00**	1.00	40	0.31

Supplementary Table 8. Parameters inferred by Random Forest for the best fitting model (Fig. 3; Raynal et al., 2019).

Parameters	Estimated value	Quantile 2.5%	Quantile 97.5%
**Effective population sizes (*Ne*) in number of diploid individuals**			
Ne M. helenor (current)	645,401	449,793	698,620
Ne M. helenor (current)	300,790	17,905	669,893
Ne M. achilles (current)	631,518	433,301	698,481
Ne M. achilles (current)	187,174	12,588	637,813
Ne M. deidamia (current)	496,636	237,820	690,466
Ne M. deidamia (current)	113,487	8,589	505,687
Ne M. helenor - M. achilles (ancestor)	409,703	87,599	673,563
Ancestral *Ne*	649,647	520,520	698,332
Shape parameter alpha	4.55	0.81	9.96
Shape parameter beta	10.20	0.57	19.59
**Time of demographic events in number of generations**			
M. helenor expansion	677,138	30,165	1,711,892
M. achilles expansion	545,804	26,027	1,559,309
M. deidamia expansion	455,822	16,735	1,695,739
Split *M. helenor - M. achilles*	1,110,618	356,427	3,451,551
Ancestral split	4,133,448	3,860,151	4,562,831
Arrest of migration *M. helenor - M. achilles*	769,233	346,007	1,270,989
**Migration (*4.N.m*)**			
M. achilles* → *M. helenor			
Migration rate	25.27	2.86	48.93
Shape parameter alpha	9.81	0.37	19.67
Shape parameter beta	10.22	0.36	19.68
M. helenor* → *M. achilles			
Migration rate	25.36	2.83	49.22
Shape parameter alpha	10.37	0.33	19.62
Shape parameter beta	9.82	0.37	19.66

Supplementary Table 9. Because the approach based on the actual numbers of SNPs does not allow the estimation of absolute values of demographic parameters (population sizes in number of individuals, age of speciation in number of generations), we provide relative values here. We thus show the parameter values of the best demographic model similarly supported in both analyses expressed relatively to the current population size of *M. helenor* set to *Ne* = 1.

**Parameters**	**Simulations conditioned on theta (expected value [95% CI])**	**Simulations conditioned on the observed number of SNPs**
Ne M. helenor (current)	1	1
Ne M. helenor (bottleneck)	0.466 [0.028 - 1.038]	0.517 [0.014 - 1.429]
Ne M. achilles (current)	0.978 [0.671 - 1.082]	0.913 [0.348 - 1.434]
Ne M. achilles (bottleneck)	0.29 [0.02 - 0.988]	0.263 [0.006 - 1.308]
Ne M. deidamia (current)	0.769 [0.368 - 1.07]	0.763 [0.174 - 1.432]
Ne M. deidamia (bottleneck)	0.176 [0.136 - 0.784]	0.169 [0.077- 0.57]
Ne M. helenor - M. achilles (ancestor)	0.635 [0.136 - 1.044]	0.69 [0.077- 1.426]
Ancestral *Ne*	1.007 [0.807 - 1.082]	0.909 [0.028 - 1.038]
**Time of demographic events**		
M. helenor expansion	1.049 [0.047 - 2.652]	0.758 [0.026 - 2.658]
M. achilles expansion	0.846 [0.04 - 2.416]	0.606 [0.023 - 1.998]
M. deidamia expansion	0.706 [0.026 - 2.627]	0.746 [0.031 - 3.954]
Split *M. helenor - M. achilles*	1.721 [0.552 - 5.348]	1.317 [0.312 - 3.26]
Ancestral split	6.404 [5.981 - 7.07]	2.977 [0.942 - 6.995]
Arrest of migration *M. helenor - M. achilles*	1.192 [0.536 - 1.969]	0.972 [0.287 - 2.082]
Arrest of migration (*M. helenor - M. deidamia*)^{ancestor}	4.745 [2.31 - 6.952]	2.745 [0.799 - 8.848]

Supplementary Table 10. Statistics describing polymorphism and genomic divergence.

	M. helenor	*M. achilles*	*M. deidamia*
Sx	0.018 ± 0.011	0.015 ± 0.010	0.005 ± 0.005
π	0.004 ± 0.003	0.003 ± 0.002	0.001 ± 0.001
Watterson's θ	0.006 ± 0.004	0.005 ± 0.003	0.002 ± 0.002
Tajima's D	-1.298 ± 0.648	-1.256 ± 0.637	-0.668 ± 0.630

	M. helenor – M. achilles	*M. helenor – M. deidamia*	*M. achilles – M. deidamia*
Sf	0.002 ± 0.004	0.025 ± 0.014	0.027 ± 0.015
Ss	0.002 ± 0.004	0.000 ± 0.001	0.000 ± 0.000
Raw divergence	0.008 ± 0.006	0.032 ± 0.016	0.032 ± 0.016
Net divergence	0.004 ± 0.005	0.029 ± 0.015	0.030 ± 0.016
F_{st}	0.305 ± 0.266	0.826 ± 0.119	0.849 ± 0.118

Number of bi-allelic positions within loci shared by the three surveyed species: n_{SNPs} = 32.429 ± 14.005

Sx : proportion of nucleotidic positions with polymorphism exclusive to a given species.

π : theta measured as the average number of pairwise differences within a given species.

Watterson's θ : theta measured as a function of the number of segregating sites within a given species.

Tajima's D : Tajima's D statistics of deviation to the standard neutral model.

Sf : proportion of nucleotidic positions with fixed difference exclusive to a given species.

Ss : theta measured as the average number of pairwise differences within a given species.

Raw divergence : average number of interspecies differences for a given pair of species.

Net divergence : average number of interspecies differences for a given pair of species by excluding within species polymorphism.

F_{st} : index of genetic differentiation.

Supplementary References

1. Tajima F. Evolutionary relationship of DNA sequences in finite populations. *Genetics* **105**, 437–460 (1983).
2. Watterson G. On the number of segregating sites in genetical models without recombination. *Theor. Popul. Biol.* **7**, 256-276. (1975).
3. Tajima F. Statistical method for testing the neutral mutation hypothesis by DNA Polymorphism. *Genetics* **123**, 585-595. (1975).
4. Nei M. *Molecular Evolutionary Genetics* (Columbia University Press, 1987).
5. Raynal, L., Marin J. M., Pudlo P., Ribatet M., Robert C. P., Estoup A., ABC random forests for Bayesian parameter inference. *Bioinformatics* **35**, 1720–1728 (2019).
