# Supplementary material for: Convergent morphology and divergent phenology promote the coexistence of Morpho butterfly species
Source: Nat Commun. 2021 Dec 13;12:7248. doi: 10.1038/s41467-021-27549-1 (PMC8668891; doi:10.1038/s41467-021-27549-1)
Supplement: Supplementary file 2 — Description of Additional Supplementary Files [file 41467_2021_27549_MOESM2_ESM.docx]

Description of Additional Supplementary Files

Title: Supplementary Video 1| Flight interaction between wild a *Morpho* butterfly and the dummy.

Description: Typical flight interaction between a patrolling *Morpho* male and the dummy butterfly showed in slow motion. Time is indicated in the top right corner. The three-dimensional position of the visitor and of the dummy is shown on the right (in blue and red respectively). Bottom panel indicates the variation in distance between visitor and dummy over time. Colour gradient shows the flight speed of the visitor *Morpho* butterfly.
