# Supplementary material for: Convergent morphology and divergent phenology promote the coexistence of Morpho butterfly species
Source: Nat Commun. 2021 Dec 13;12:7248. doi: 10.1038/s41467-021-27549-1 (PMC8668891; doi:10.1038/s41467-021-27549-1)
Supplement: Supplementary file 4 — Reporting Summary [file 41467_2021_27549_MOESM4_ESM.pdf]

nature research

Corresponding author(s): Camille Le Roy

Last updated by author(s): Nov 1, 2021

Reporting Summary

Nature Research wishes to improve the reproducibility of the work that we publish. This form provides structure for consistency and transparency in reporting. For further information on Nature Research policies, see our Editorial Policies and the Editorial Policy Checklist.

Statistics

For all statistical analyses, confirm that the following items are present in the figure legend, table legend, main text, or Methods section.

n/a	Confirmed	
☐	☒	The exact sample size (n) for each experimental group/condition, given as a discrete number and unit of measurement
☐	☒	A statement on whether measurements were taken from distinct samples or whether the same sample was measured repeatedly
☐	☒	The statistical test(s) used AND whether they are one- or two-sided<br>*Only common tests should be described solely by name; describe more complex techniques in the Methods section.*
☐	☒	A description of all covariates tested
☐	☒	A description of any assumptions or corrections, such as tests of normality and adjustment for multiple comparisons
☐	☒	A full description of the statistical parameters including central tendency (e.g. means) or other basic estimates (e.g. regression coefficient) AND variation (e.g. standard deviation) or associated estimates of uncertainty (e.g. confidence intervals)
☐	☒	For null hypothesis testing, the test statistic (e.g. F, t, r) with confidence intervals, effect sizes, degrees of freedom and P value noted<br>*Give P values as exact values whenever suitable.*
☒	☐	For Bayesian analysis, information on the choice of priors and Markov chain Monte Carlo settings
☒	☐	For hierarchical and complex designs, identification of the appropriate level for tests and full reporting of outcomes
☒	☐	Estimates of effect sizes (e.g. Cohen's d, Pearson's r), indicating how they were calculated

Our web collection on statistics for biologists contains articles on many of the points above.

Software and code

Policy information about availability of computer code

Data collection	Flight kinematic data were obtained using the open source Matlab routine DLTdv8, available at https://biomech.web.unc.edu/dltdv/.<br>Genomic analyses were performed using the statistical analysis platform DILS 1.0.0, available at https://eep.univ-lille.fr/en/productions-2/dils-software/. Source codes to deploy DILS can be freely used from GitHub: https://github.com/popgenomics/DILS_web.<br>Reads were aligned using Stacks (version 2.5).
Data analysis	All experimental data were analysed using the software R 3.6.0. We used the R packages: Rcapture (version 1.4.3), abcrf (version 1.8.1), rgl (version 0.106.8), multcomp (version 1.4.17), ggplot2 (version 3.3.3).

For manuscripts utilizing custom algorithms or software that are central to the research but not yet described in published literature, software must be made available to editors and reviewers. We strongly encourage code deposition in a community repository (e.g. GitHub). See the Nature Research guidelines for submitting code & software for further information.

Data

Policy information about availability of data

All manuscripts must include a data availability statement. This statement should provide the following information, where applicable:

- Accession codes, unique identifiers, or web links for publicly available datasets
- A list of figures that have associated raw data
- A description of any restrictions on data availability

All data supporting the findings of this study are provided as a Source Data file. Source data are provided with this paper. The RNA-seq data generated in this study have been deposited to National Center for Biotechnology Information under accession number PRJNA739839 (https://www.ncbi.nlm.nih.gov/).

Field-specific reporting

Please select the one below that is the best fit for your research. If you are not sure, read the appropriate sections before making your selection.

☐ Life sciences ☐ Behavioural & social sciences ☒ Ecological, evolutionary & environmental sciences

For a reference copy of the document with all sections, see nature.com/documents/nr-reporting-summary-flat.pdf

Ecological, evolutionary & environmental sciences study design

All studies must disclose on these points even when the disclosure is negative.

Study description	Quantification of reproductive interference, genetic exchanges and temporal niche dimension among closely-related and phenotypically similar Morpho butterfly species.
Research sample	Reproductive interference was assessed by investigating the behavioural response of wild butterflies to dummies.<br>Ten dummies were each tested during 4 sunny days from 9 a.m. to 2 p.m. (i.e. 4 replicates of 5 hours per dummy). This resulted in 40 days of experiment over which each dummy was left fluttering on the river bank for a combined duration of 20 hours. > 2,700 butterfly responses to dummies were studied in total.<br>Every 2 weeks, stereoscopic high-speed videography system was set up to quantified the flight behaviour of patrolling males around dummies (5 replicates stereoscopic videography in total). This resulted in 14 flight interactions studied for each dummy sex (n = 28) in the species M. achilles.<br>Patrolling time among species was measured through mark-recapture experiment: number of individuals captured in M. helenor - n = 92; M. achilles - n = 121; M. deidamia - n = 48; M. menelaus - n = 34.<br>DNA extraction was performed on 31 individuals (M. achilles - n = 13, M. helenor - n = 10 and M. deidamia - n = 8).<br>We studied the population of Morpho butterflies living along the Shilcayo river near the city of Tarapoto in North Peru (exact location of the experiment: 06°27'14.364" S, 76°20'45.852" W, ca. 300 m a.s.l.).
Sampling strategy	Sample size for encounters with dummies depended on the abundance of individual butterflies patrolling in the wild at the study site. Performing 40 days of experiment provided an sufficient sample size (> 2,700 butterfly responses to dummies studied in the entire experiment) to investigate discriminative behaviour among sympatric Morpho species.<br>Sample size for mark-recapture depended on capture effort. We performed 17 days of capture (5 hours in each day) to measure patrolling time in a sufficient number of individuals (M. helenor - n = 92; M. achilles - n = 121; M. deidamia - n = 48; M. menelaus - n = 34).<br>For DNA extraction, about 10 individuals per species was considered enough to cover intraspecific genetic variation.
Data collection	During the dummy experiment, the dummy was continuously filmed using a gopro camera and monitored by Camille Le Roy who recorded the timing of responses displayed by wild butterflies. Timing was recorded with a timer, a pen and a paper sheet. For the mark-recapture, butterflies were captured with hands-nets, identified at the species level, and numbered on their dorsal wing surface using a black marker.<br>For the genomic analyses, RAD library preparation and sequencing were performed at the MGX-Montpellier GenomiX platform (Montpellier, France).
Timing and spatial scale	The mark-recapture study was planned such as sampling several tens of individuals in the different sympatric Morpho species. Twenty-two days in total (including rainy days cancelling butterfly activity) were needed to obtain suitable sample size. Timing for the dummy experiment was planned such as performing 4 replicates of 5 hours for each of the 10 tested dummies (under sunny condition only). This was achieved over a two months period (late July 2019 to early October 2019). No gap larger than 4 days occured in the data collection. All the experiments were performed in the same field locality (riverbank location: 06°27'14.364" S, 76° 20'45.852" W). The overall field study lasted from July to October 2019.
Data exclusions	No data were excluded from the analyses.
Reproducibility	The ten dummies were each tested during 4 sunny days from 9 a.m. to 2 p.m. (i.e. during 5 hours). The 4 replicates of each dummy were performed in a random order (dummies were randomly attributed to each day of experiment).
Randomization	Dummies were randomly attributed to each day of experiment. Mark-recapture data suggested a very low rate of individuals passing through the site several times per day (mean percentage of recapture within the same day = 0.95%), thus limiting potential pseudoreplication within each dummy replicate.
Blinding	We are aware that blinding is very important for behavioural experiment, nevertheless our dummy experiment was conducted in the wild, behaviour directly quantified in-situ, preventing the application of full blinding.

Did the study involve field work? ☒ Yes ☐ No

Field work, collection and transport

Field conditions	The experiments were performed on a river bank of the Peruvian Amazonia, under sunny condition only. Mean temperature during the experiment = 26 +/- 2C°. Mean relative humidity = 80 +/- 9% (measured in shadow).

Location	Both the capture-recapture and the dummy experiment were performed at the exact same location, on the bank of the Shilcayo river (06°27'14.364" S, 76°20'45.852" W, ca. 300 m a.s.l.), near the city of Tarapoto (North Peru).
Access & import/export	Exportation of the Individual butterflies (for DNA extraction and genomic analyses) was done in compliance with the Peruvian SERFOR (Servicio Nacional Forestal y de Fauna Silvestre) using the research permit 373-2017-SERFOR-DGGSPFFS issued on October 20th 2017 for a 4 years validity period.
Disturbance	The dummy experiment had no disturbing effect on the butterflies. All butterflies captured during the mark-recapture experiment were released at same place of the capture after being marked. 31 individuals were captured and killed for DNA extraction. 10 additional individuals were killed to build the dummy butterflies. Morpho butterfly species are not endangered.

Reporting for specific materials, systems and methods

We require information from authors about some types of materials, experimental systems and methods used in many studies. Here, indicate whether each material, system or method listed is relevant to your study. If you are not sure if a list item applies to your research, read the appropriate section before selecting a response.

Materials & experimental systems

n/a	Involved in the study
☒	☐ Antibodies
☒	☐ Eukaryotic cell lines
☒	☐ Palaeontology and archaeology
☐	☒ Animals and other organisms
☒	☐ Human research participants
☒	☐ Clinical data
☒	☐ Dual use research of concern

Methods

n/a	Involved in the study
☒	☐ ChIP-seq
☒	☐ Flow cytometry
☒	☐ MRI-based neuroimaging

Animals and other organisms

Policy information about studies involving animals; ARRIVE guidelines recommended for reporting animal research

Laboratory animals	The study did not involve laboratory animals.
Wild animals	The study involved observations in natura of Morpho butterflies (M. helenor, M. achilles, M. deidamia and M. menelaus, all males). For the mark-recapture study, butterflies were captured using hand-net, marked on the dorsal wings with a marker, and released afterward at same place of the capture. Individuals collected for DNA extraction and for building of the dummies were carried in entomological envelopes and killed by placing them in a -20°C freezer. The age of the studied individuals is unknown.
Field-collected samples	Individuals collected for DNA extraction were killed by placing them in a −20°C freezer near the field study site. Thoraxes of the collected butterflies were then stored in dimethylsulfoxide (DMSO) in a -80°C freezer awaiting DNA extraction.
Ethics oversight	No ethical approval was required for invertebrates.

Note that full information on the approval of the study protocol must also be provided in the manuscript.
